# Changes in Skeletal Muscle Protein Metabolism Signaling Induced by Glutamine Supplementation and Exercise

**DOI:** 10.3390/nu15224711

**Published:** 2023-11-07

**Authors:** Carlos Flores Rodrigues Junior, Gilson Masahiro Murata, Frederico Gerlinger-Romero, Renato Tadeu Nachbar, Gabriel Nasri Marzuca-Nassr, Renata Gorjão, Kaio Fernando Vitzel, Sandro Massao Hirabara, Tania Cristina Pithon-Curi, Rui Curi

**Affiliations:** 1 Department of Physiology and Biophysics, Institute of Biomedical Sciences, University of São Paulo, São Paulo 05508-220, Brazil; carlosflores.uem@hotmail.com (C.F.R.J.); tania.pithon-curi@cruzeirodosul.edu.br (T.C.P.-C.); rui.curi@cruzeirodosul.edu.br (R.C.); 2 Divisions of Nephrology and Molecular Medicine, LIM-29, Department of Medicine, University of São Paulo, São Paulo 05508-220, Brazil; gilmasa@gmail.com; 3 School of Medicine, University of Mogi das Cruzes, Mogi das Cruzes 08780-911, Brazil; fredericoromero@umc.br; 4 Departamento de Ciencias de la Rehabilitación, Facultad de Medicina, Universidad de La Frontera, Temuco 4811230, Chile; gabriel.marzuca@ufrontera.cl; 5 Interuniversity Center for Healthy Aging (Code RED21993), Talca 3460000, Chile; 6 Interdisciplinary Post-graduate Program in Health Sciences, Universidade Cruzeiro do Sul, São Paulo 01506-000, Brazil; sandro.hirabara@cruzeirodosul.edu.br; 7 School of Health Sciences, Massey University (University of New Zealand), Auckland 0745, New Zealand; k.vitzel@massey.ac.nz; 8 Butantan Institute, São Paulo 05585-000, Brazil

**Keywords:** physical exercise, hypertrophy, pS6, proteasome, 4E-BP-1, 26S proteasome

## Abstract

Aim: To evaluate the effects of resistance exercise training (RET) and/or glutamine supplementation (GS) on signaling protein synthesis in adult rat skeletal muscles. Methods: The following groups were studied: (1) control, no exercise (C); (2) exercise, hypertrophy resistance exercise training protocol (T); (3) no exercise, supplemented with glutamine (G); and (4) exercise and supplemented with glutamine (GT). The rats performed hypertrophic training, climbing a vertical ladder with a height of 1.1 m at an 80° incline relative to the horizontal with extra weights tied to their tails. The RET was performed three days a week for five weeks. Each training session consisted of six ladder climbs. The extra weight load was progressively increased for each animal during each training session. The G groups received daily L-glutamine by gavage (one g per kilogram of body weight per day) for five weeks. The C group received the same volume of water during the same period. The rats were euthanized, and the extensor digitorum longus (EDL) muscles from both hind limbs were removed and immediately weighed. Glutamine and glutamate concentrations were measured, and histological, signaling protein contents, and mRNA expression analyses were performed. Results: Supplementation with free L-glutamine increased the glutamine concentration in the EDL muscle in the C group. The glutamate concentration was augmented in the EDL muscles from T rats. The EDL muscle mass did not change, but a significant rise was reported in the cross-sectional area (CSA) of the fibers in the three experimental groups. The levels of the phosphorylated proteins (pAkt/Akt, pp70S6K/p70S6K, p4E-BP1/4E-BP1, and pS6/S6 ratios) were significantly increased in EDL muscles of G rats, and the activation of p4E-BP1 was present in T rats. The fiber CSAs of the EDL muscles in T, G, and GT rats were increased compared to the C group. These changes were accompanied by a reduction in the 26 proteasome activity of EDL muscles from T rats. Conclusion: Five weeks of GS and/or RET induced muscle hypertrophy, as indicated by the increased CSAs of the EDL muscle fibers. The increase in CSA was mediated via the upregulated phosphorylation of Akt, 4E-BP1, p70S6k, and S6 in G animals and 4E-BP1 in T animals. In the EDL muscles from T animals, a decrease in proteasome activity, favoring a further increase in the CSA of the muscle fibers, was reported.

## 1. Introduction

Skeletal muscle hypertrophy and strength gains are desired by elite athletes, patients with rehabilitation-induced atrophy, and older individuals with limited mobility due to muscle weakness-related injuries. Researchers have used electrical stimulation and interventions (pharmacological and physiological) to discover effective protocols to induce muscle hypertrophy [1,2,3]. Progressive resistance exercise training has been employed to enhance the mass and strength of skeletal muscles [4]. Rat resistance exercise has proven to resemble human resistance exercise training protocols, so various resistance training protocols are used in studies on skeletal muscle mass gain [2,3,5,6,7,8,9,10,11,12,13].

Protein metabolism (synthesis and degradation) in skeletal muscle is mediated via several signaling molecules that regulate transcriptional and post-transcriptional steps [14]. One of the primary factors in the control of protein synthesis is Akt protein, which induces the activation of hypertrophy in vivo, as reported in the skeletal muscle of transgenic mice overexpressing this signaling molecule [15]. Akt activation for two weeks in adult animals raised the expression of signaling proteins involved in protein synthesis, such as mammalian rapamycin (mTOR) and p70S6K, increasing skeletal muscle cell size by two-fold and the number of muscle fibers [15].

Under conditions of skeletal muscle atrophy due to disuse, fasting, denervation, cachexia, burns, or renal failure [16,17,18,19,20,21,22,23], Akt activation is suppressed [24]. mTOR promotes 4E-BP1 (eIF4E-binding protein-1) and ribosomal protein S6 kinase 1 (S6K1) phosphorylation and initiation of protein synthesis [25,26]. Non-phosphorylated 4E-BP1 and eIF4E form an inactive complex that inhibits translation; in opposition, the phosphorylated 4E-BP1 allows eIF4E to bind to eIF4G, forming an active complex [21]. The mTOR inhibits 4E-BP1 (a negative regulator of eIF-4E) [21,27,28]. Insulin and amino acids (leucine and arginine) induce 4E-BP1 phosphorylation via mTOR signaling [28].

Ribosomal protein S6 regulates the selection of mRNAs for translation [29] and upregulates protein synthesis [29]. Leucine activates S6 by promoting its phosphorylation [28,30,31]. Leucine inhibits activation of atrogin-1 or MAFbx (muscular atrophy F-box) and MuRF-1 (muscle ring finger-1), reducing skeletal muscle mass loss via Akt. Akt phosphorylates FOXO, leading to its translocation from the nucleus to the cytosol [32,33]. In the cytosol, FOXO inactivates and inhibits the activation of MAFbx/atrogin-1 and MuRF-1 [32,33,34]. MAFbx/atrogin-1 and MuRF-1 belong to the ubiquitin ligase family. This pathway is stimulated by skeletal muscle loss resulting from fasting, hindlimb suspension, and immobilization [32,35].

Glutamine upregulates the mTOR pathway involved in skeletal muscle hypertrophy; it increases leucine uptake via HeLa cells [36,37,38,39,40]. Lambertucci et al. (2012) reported decreased muscle glutamine content and fiber cross-sectional area (CSA) in diabetic rats [41]. The diabetic rats also exhibited downregulated gene expression of regulatory proteins involved in protein synthesis and upregulated expression of protein degradation-associated signaling molecules. The authors also reported that supplementing diabetic rats with glutamine gradually enhances muscle glutamine content toward the control value and alleviates the skeletal muscle mass decrease. Glutamine pretreatment attenuates 24 h fasting-induced skeletal muscle atrophy. This latter finding was associated with the activation of protein synthesis signaling p-RPS6Ser240/244 and Akt-mTOR [21]. We reported that glutamine supplementation during an exhaustion test results in exercising at a more elevated second ventilatory threshold, maximal oxygen consumption percentage, and blood lactate concentrations, accompanied by decreased plasma levels of skeletal muscle damage markers in elite triathletes [42].

Herein, we described findings on the effects of glutamine supplementation (GS) associated or not with resistance exercise training (RET) on expression and activation of protein synthesis (pAkt/Akt, pp70S6K/p70S6K, p4E-BP1/4E-BP1, pS6/S6) signaling in the extensor digitorum longus (EDL) muscle of adult male rats. Evidence is presented that glutamine participates in the regulation of protein synthesis, protein content, and myofibrillar CSAs in rat EDL muscle, and these effects are observed even when not associated with exercise training.

## 2. Results

**Figure 1:**
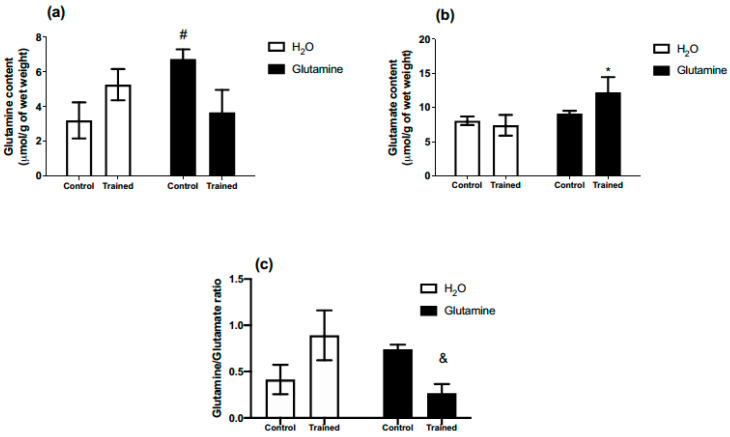
Effects of L-glutamine supplementation (daily doses of 1 g/kg body weight for five weeks) associated or not with resistance exercise training on the concentrations of glutamine (**a**) and glutamate (**b**) in the EDL muscle. (**c**) Glutamine/glutamate ratio in EDL muscle. The results are presented as the mean ± SEM (standard error of the mean) and were compared using a two-way ANOVA test and the Bonferroni post-test (n = 5 to 6 animals per group). * *p* = 0.0422 versus T (exercise, hypertrophy resistance training exercise protocol). ^#^ *p* = 0.0447 versus C (Control, no exercise). ^&^ *p* = 0.0286 versus T (exercise, hypertrophy resistance training exercise protocol).

All rats were able to complete the five-week RET protocol successfully. GS increased glutamine concentration in the EDL muscles of the C group by ~80% (Figure 1a). Moreover, glutamate content in the EDL muscles of T rats was augmented by ~50% (Figure 1b). The glutamine/glutamate ratio was lower in the supplemented trained group (GT) in comparison to the non-supplemented trained group (T) (Figure 1c).

**Figure 2:**
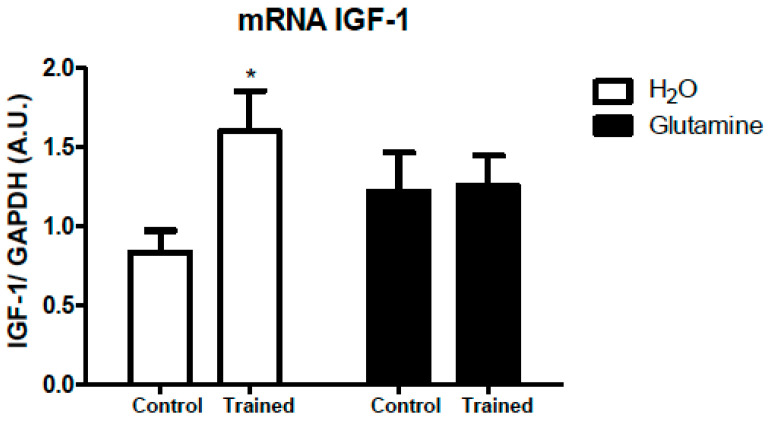
Effects of L-glutamine supplementation (daily doses of 1 g/kg body weight for five weeks) associated or not with resistance exercise training on the expression of IGF-1 mRNA in the EDL muscle. The results are presented as the mean ± SEM (standard error of the mean) and were compared using a two-way ANOVA test and the Bonferroni post-test (n = 5 to 6 animals per group).* *p* < 0.05 vs. C. Control, no exercise (C); exercise, hypertrophy resistance training exercise protocol (T); no exercise, supplemented with glutamine (G); and exercise and supplemented with glutamine (GT).

The IGF-1 mRNA expression in EDL muscles of T rats was downregulated by 50% (Figure 2).

**Figure 3:**
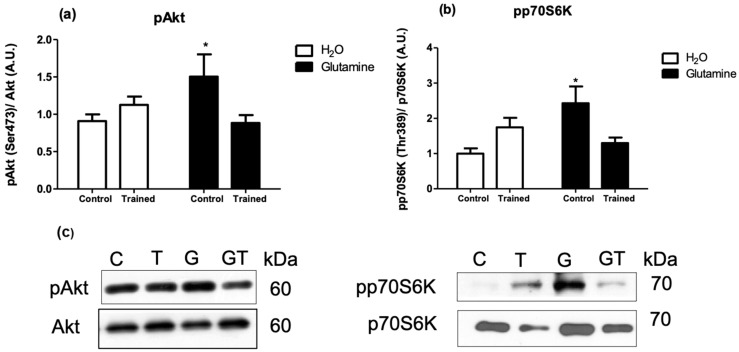
Effects of L-glutamine supplementation (daily doses of 1 g/kg body weight for five weeks) associated or not with resistance exercise training on the levels of phosphorylated Akt-1 (**a**) and p70S6K (**b**) in the EDL muscle. Representative bands of the proteins are shown in (**c**). The results are presented as the mean ± SEM (standard error of the mean) and were compared using a two-way ANOVA and the Bonferroni post-test (n = 8 to 9 animals per group). * *p* < 0.05 vs. C. Control, no exercise (C); exercise, hypertrophy resistance training exercise protocol (T); no exercise supplemented with glutamine (G); and exercise and supplemented with glutamine (GT).

**Figure 4:**
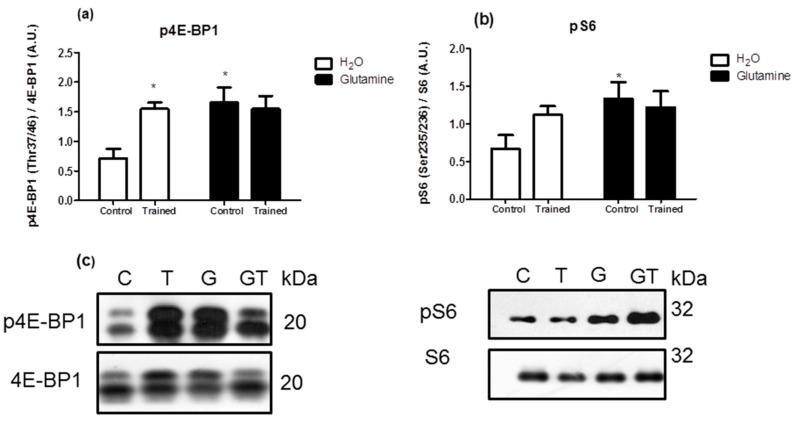
Effects of L-glutamine supplementation (daily doses of 1 g/kg body weight for five weeks) associated or not with resistance exercise training on the levels of phosphorylated P-4E-BP1 (**a**) and pS6 (**b**) in the EDL muscle. Representative bands of the proteins are shown in (**c**). The results are presented as the mean ± SEM (standard error of the mean) and were compared using a two-way ANOVA and the Bonferroni post-test (n = 8 to 9 animals per group). * *p* < 0.05 vs. C. Control, no exercise (C); exercise, hypertrophy resistance training exercise protocol (T); no exercise, supplemented with glutamine (G); and exercise and supplemented with glutamine (GT).

The levels of the phosphorylated proteins (pAkt/Akt, pp70S6K/p706K, p4E-BP1/4E-BP1, and pS6/S6 ratios) were significantly increased in EDL muscles of G rats, attaining levels of approximately 150%, 200%, 150%, and 200% greater than in C rats, respectively (Figure 3a,b and Figure 4a,b).

**Figure 5:**
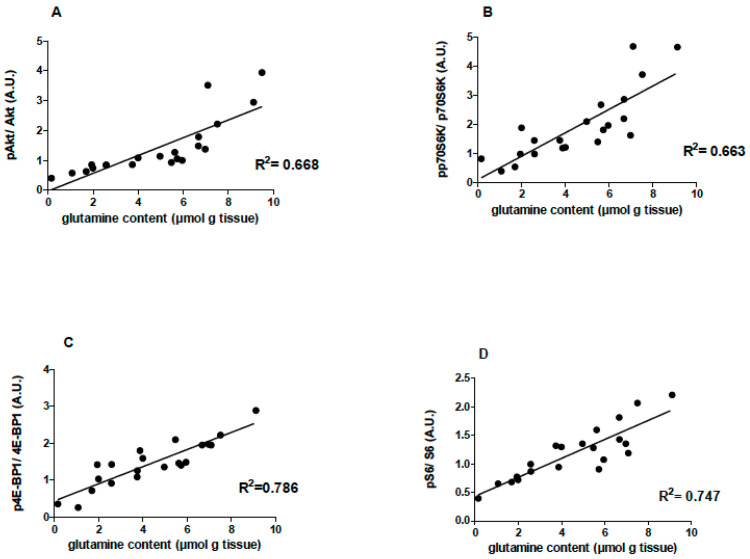
Effects of L-glutamine supplementation (daily doses of 1 g/kg body weight for five weeks) associated or not with resistance exercise training on the correlation between muscle glutamine content and pAkt (n = 20), pp70s6k (n = 21), p4E-BP1 (n = 21) and pS6 (n = 21) levels in the EDL muscle of the animals. (**A**) Correlation between EDL muscle glutamine concentration and pAkt/Akt. (**B**) Correlation between EDL muscle glutamine concentration and ppP70S6k/pP70S6k. (**C**) Correlation between EDL muscle glutamine concentration and p4E-BP1/4E-BP1. (**D**) Correlation between EDL muscle glutamine concentration and pS6/S6.Control, no exercise (C); exercise, hypertrophy resistance training exercise protocol (T); no exercise supplemented with glutamine (G); and exercise and supplemented with glutamine (GT).

A positive correlation between the EDL muscle glutamine concentration and pAkt (R^2^ = 0.668), ppP70S6k (R^2^ = 0.663), p4E-BP1 (R^2^ = 0.786), and pS6 (R^2^ = 0.747) levels was reported (Figure 5).

**Figure 6:**
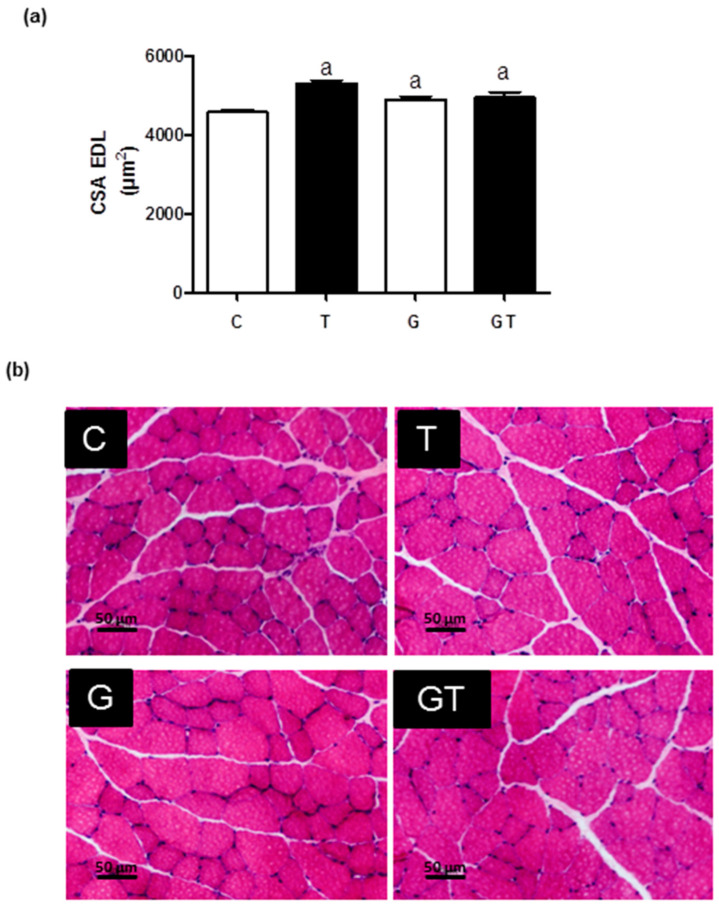
Effects of L-glutamine supplementation (daily doses of 1 g/kg body weight for five weeks) associated or not with resistance exercise training on the cross-sectional area (CSA) of muscle fibers of the EDL. (**a**) CSA (μm^2^) Mean ± SEM (standard error of the mean), and (**b**) muscle fibers CSA images (20×) obtained via optical microscopy after hematoxylin and eosin staining (HE). One thousand fibers were analyzed per group. Significant differences were reported using 95% confidence interval, as described in Materials and Methods (n = 6 animals per group). Control, no exercise (C); exercise, hypertrophy resistance training exercise protocol (T); no exercise supplemented with glutamine (G); and exercise and supplemented with glutamine (GT).

The fiber CSAs of the EDL muscles (Figure 6) in the T, G, and GT groups were increased by 20% compared to the C group.

**Figure 7:**
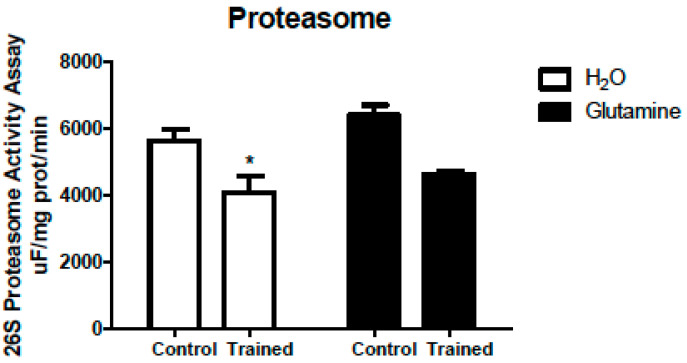
Effects of L-glutamine supplementation (daily doses of 1 g/kg body weight for five weeks) associated or not with resistance exercise training on the activity of the 26S proteasome in the EDL muscle. The results are presented as the mean ± SEM (standard error of the mean) and were compared using two-way ANOVA and the Bonferroni post-test. (n = 5 animals per group). * *p* < 0.05 vs. C. Control, no exercise (C); exercise, hypertrophy resistance exercise training protocol (T); no exercise supplemented with glutamine (G); and exercise and supplemented with glutamine (GT).

These changes reported were accompanied by a 30% reduction in 26S proteasome activity (Figure 7).

## 3. Discussion

The effects of GS or RET on protein synthesis signaling have been reported previously; however, the underlying molecular basis for these effects has yet to be established. Herein, we investigated in rats whether GS and RET can modulate signaling pathways of protein synthesis (pAkt/Akt, pp70S6K/p706K, p4E-BP1/4E-BP1, and pS6/S6 ratios) in the EDL muscle. GS without RET increased glutamine concentrations in the EDL muscles. The GT animals had elevated EDL muscle glutamate concentrations. Glutamine generates glutamate in a reaction catalyzed via phosphate-dependent glutaminase [43,44,45]. Thus, most of the glutamine was utilized to form glutamate in the EDL muscle from the GT group.

The EDL muscle contains predominantly type IIb fibers, which exhibit rapid contractions, are glycolytic, and are more responsive to RET [46]. The RET protocol employed in this study stimulated protein synthesis, resulting in morphological changes like increased fiber CSA in the short term. The morphophysiological changes during hypertrophy are well documented in RET protocols, such as climbing [4,8,47,48,49].

Previous work reported that changes in p4E-BP1 and pS6 do affect protein synthesis [33]. We reported on the activation of p4E-BP1 in exercise-trained animals. The upregulated protein synthesis pathway most likely accounts for the augmented CSA of the muscle fibers.

The Akt signaling regulates protein synthesis and controls FOXO expression [18,48], regulating the expression of E3 ligases MAFbx/atrogin-1 and MuRF-1, involved in protein degradation via the ubiquitin-proteasome system (UPS) that designates target proteins for degradation. MuRF-1 and atrogin-1 reportedly increase in skeletal muscles under protein catabolic conditions such as fasting, cancer, diabetes, and muscle immobilization [32]. The enteral infusion of glutamine in humans caused a reduction in the content of ubiquitin mRNA in the intestine [50]. However, the calpain and cathepsin mRNA levels were not changed under these conditions, and an increase in protein synthesis was observed, as measured via mass spectrometry. The authors postulated that glutamine stimulates protein synthesis and attenuates proteolysis to maintain protein homeostasis in the human intestine [50]. Herein, at the end of the five-week program, there was a significant decrease in UPS activity, possibly contributing to avoiding a massively decreased protein degradation to preserve skeletal muscle mass. The GS did not alter the UPS activity..

Rats treated with dexamethasone and fed a diet rich in whey protein enriched with glutamine exhibited a high skeletal muscle protein synthesis compared to non-supplemented ones [51]. Others have also used climbing-based physical training twice daily for 12 weeks and did not detect changes in GSK3-beta, Akt, or p70SK protein content in the *plantaris* muscle from Wistar rats [52].

Several studies also reported that amino acids associated with different experimental models lead to increased muscle protein synthesis. One study supports that muscle cells’ intrinsic ability to respond to GS is preserved in healthy older women [53]. Leucine induces protein synthesis in the murine EDL muscles, increasing the phosphorylation of mTOR and p70S6, and these proteins play a significant role in protein synthesis [54].

In pigs fed a low-protein diet, leucine supplementation increases the rate of protein synthesis in the gastrocnemius and the *latissimus dorsi* muscles and upregulates mTOR, 4E-BP1, and S6 phosphorylation [55]. Another study in pigs supplemented with leucine, isoleucine, and valine reported that only leucine supplementation increases the protein synthesis rate compared with saline [56]. This latter study also demonstrated increased phosphorylation of key proteins involved in protein synthesis (e.g., 4E-BP1 and S6) in animals infused with leucine for 60 min. Leucine supplementation combined with 8-week RET increased the differentiation of satellite cells and myofiber CSAs in rats [57].

Arginine also has anabolic effects. Arginine and glutamine improved total body lean mass in a small sample of healthy-aged humans [58]. In pigs supplemented with arginine for seven days, the phosphorylation of proteins involved in protein synthesis, such as mTOR, 4E-BP1, and eIF4G-eIF4E, and the *latissimus dorsi* muscle weight were increased [59].

GS increased EDL muscle protein synthesis signaling phosphorylation (pAkt/Akt, pp70SK1/p70S6K, p4E-BP1/4E-BP1, and pS6/S6 ratios). The EDL muscle IGF-1 mRNA content was raised in the T group, but this was not found in the GT. A higher intramuscular glutamine concentration generated via exercise-induced proteolysis and glutamine supplementation may affect leucine entering the skeletal muscle [45]. Protein synthesis does not occur properly when required amino acids are not provided in the needed amount to activate the mTOR complex [60].

Clarification on the entire mechanism involved is a limitation of the present study. GS and RET for a more extended period would make the changes herein reported more pronounced. Western blotting assay is, per se, a questionable protein quantification technique. Finding the most appropriate protein sample amount is always challenging to avoid overloading. So, the protein results analysis should consider this protein quantity limitation.

A correlation of muscle glutamine concentration with the activation of essential signaling proteins involved in protein synthesis (pAkt/Akt, pp70SK1/p70S6K, p4E-BP1/4E-BP1, and pS6/S6 ratios) was found (Figure 5). A study with gastrocnemius muscles from rats infused with glutamine reported an inhibitory effect on muscle protein breakdown by measuring phenylalanine incorporation into proteins [61]. The findings suggest a correlation between skeletal muscle mass loss and low intramuscular glutamine concentrations and a positive correlation between muscle glutamine and the protein synthesis rate, indicating that intramuscular glutamine concentration plays a role in controlling skeletal muscle mass [61]. Another study with gastrocnemius muscles from rats in the presence or absence of insulin also detected a positive correlation between protein synthesis and intramuscular glutamine concentration [62]. We reported tissue L-glutamine levels per se positively associated with the protein content in skeletal muscle [63]. Low levels of plasma and skeletal muscle glutamine are reported in severe illness. Glutamine deficiency may disrupt mitochondrial integrity, impairing cell function [64].

Glutamine metabolism restores mTORC1 activity after prolonged amino acid starvation through autophagy [65]. Our group reported that GS pre-treatment attenuates skeletal muscle atrophy induced by 24 h fasting [21]. GS increased the CSA of EDL muscle fibers, as reported in muscle overload-induced hypertrophy [66]. For the first time, we also described that oral GS for 30 days improves the strength and power of knee muscles in association with improved glycemia control and concomitant boost of plasma antioxidant capacity of exercising-aged women [67]. The observations of the studies by others [31] and ours indicate a positive correlation between muscle glutamine content and several phosphorylated protein synthesis signaling molecule levels.

## 4. Materials and Methods

### 4.1. Animals

Male Wistar rats (110–150 g body weight) were provided by the Department of Physiology and Biophysics, Institute of Biomedical Sciences—University of São Paulo (ICB-USP) and maintained at 23 ± 2 °C and 12/12 h dark/light cycle. The rats had free access to food (Nuvilab CR1, Nuvital Nutrientes Ltd., Curitiba, Brazil) and water. The Ethical Committee of the ICB-USP (Permit Number: 012-125-02) approved this study. The animals were equally and randomly separated into four groups: (1) control, no exercise (C); (2) exercise, hypertrophy resistance exercise training protocol (T); (3) no exercise, supplemented with glutamine (G); and (4) exercise and supplemented with glutamine (GT).

### 4.2. Resistance Exercise Training (RET) Protocol

The rats climbed a 1.1 m ladder (80° incline) with extra weights tied to their tails. The RET was performed three days per week on non-consecutive days for five weeks. Each training session consisted of six ascents up the ladder, with an average of 12 min. The extra weight load progressively increased for each animal during each training session. The ladder length allowed the animals to make 8–12 dynamic movements per climb with a 1 min rest interval between the repetitions and a 2 min rest between the six sets.

Three days following adaptation, the rats initiated the high-intensity progressive RET program. During the first four ladder ascents, the rats climbed the ladder carrying weights corresponding to the maximal carrying capacity of the previous climb (50%, 75%, 90%, and 100%, respectively). Additional 30 g loads were successively added to the tail for the subsequent ladder climbs, achieving a new maximal carrying capacity [4]. As encouragement during the training, we used an occasional hand prod at the base of the animal’s tail. No food reward, restricted food intake, or use of unnatural incentives (e.g., cold water, forced air, or electrical stimulation) was required for the rats to exercise. Others used similar strategies [4,13].

### 4.3. Glutamine Supplementation (GS)

An aqueous glutamine (SigmaAldrich, St. Louis, MO, USA) solution was prepared before use. The rats were supplemented daily with L-glutamine by gavage (one g per Kg body weight) for five weeks. The C group received the same volume of water. Others administered the same dose of glutamine for shorter periods in Wistar rats and reported augmented concentrations of this amino acid in the plasma and skeletal muscle [21,41,68].

### 4.4. Determination of Glutamine and Glutamate Contents in EDL Muscle

The EDL muscles were removed, frozen in liquid nitrogen, and stored at −80 °C until analysis. As previously described, sample preparations and measurements of glutamine and glutamate were carried out [21,41].

Using a Polytron, the tissues were homogenized in Tris EDTA (1:10 *w*/*v*; mass ratio to the volume of Tris-EDTA buffer). Protein precipitation was performed by mixing the samples with 10% perchloric acid (PCA) at a ratio of 1:1 (*v*/*v*). Immediately after precipitation with PCA, pH neutralization was carried out by adding about 1/3 of the sample volume with 2M KOH [21,41]. The volume of KOH used was sufficient to bring the pH of the samples to approximately 6.5, as indicated by the green color of the universal pH indicator [21,41]. The samples were centrifuged at 3000× *g* for 10 min at 4 °C. For the glutamine and glutamate assay, 20 µL of the supernatant was used, and each experiment was performed in triplicate [21,41]. The amounts of L-glutamine and/or glutamate were monitored by measuring the absorbance at 340 nm on a microplate ELISA reader (Biorad Benchmark Microplate Reader 340–750 nm UV/VIS, Hercules, CA, USA), which corresponds to NADH formation.

### 4.5. Histological Analysis: Fiber Cross-Sectional Area (CSA) Determination of the EDL Muscles

The rats were killed 48 h after the last session of the RET. The EDL muscles were carefully harvested, snap-frozen in isopentane, and stored at −80 °C [21,22]. Like previous studies [21,22], we used a cryostat to cut sections from the mid-belly region of the muscle medial portion. The tissue sections were stained with hematoxylin and eosin (HE) to examine the CSAs of the fibers. We examined the CSAs at 20× magnification and used the Image Pro-Plus (Media Cybernetic, Rockville, MD, USA) for analysis. We calculated the mean CSAs by measuring the circumference of 100 adjacent fibers from the center of each cross-section, 1000 fibers per muscle tissue [21,22]. A single observer blinded to the rat treatment conducted all analyses.

### 4.6. Real-Time PCR Analysis of IGF-1 and GAPDH mRNA Expression

The mRNA expression was measured via real-time PCR using the ROTOR GENE 3000 apparatus (Corbett Research, Mortlake, NSW, Australia). The total muscle RNA was obtained using Trizol reagent (Invitrogen Life Technologies, Rockville, MD, USA). The EDL muscle was lysed using 1 mL Trizol reagent, and the total RNA was extracted and stored as previously described [69,70].

The cDNA probes were synthesized using 2 µg of total RNA and a mixture containing 146 ng random primers, 200 U reverse transcriptase (Invitrogen Life Technologies), 5× reaction buffer (50 mM Tris–HCl, pH 8.0; 75 mM KCl; 3 mM MgCl_2_), 5 mM DTT, and 500 µM dNTP, in a final volume of 20 µL as previously reported [69,70]. The cDNA was stored at −20 °C, and one µg was used in the real-time PCR assay. The reaction mixture contained 100 µM dNTPs, 10× reaction buffer (10 mM Tris–HCl, 50 mM KCl, 2 mM MgCl_2_), and 1 U Taq DNA polymerase (Invitrogen Life Technologies), and 0.1 µM of each primer (sense and antisense). SYBR GREEN (diluted 1:1000) (Invitrogen Life Technologies) was used as a fluorescent dye [69,70]. The primer sequences were designed using the information contained in the GenBank database from the National Center for Biotechnology Information (NCBI). The sense and antisense sequences and the annealing temperatures for IGF-1 are presented in Table 1.

The RT-PCR cycle threshold (CT) was assessed in duplicate for each sample. The CT values are equal to the PCR cycle number and represent the intensity of fluorescence emitted via the amplification product of the target gene. They are also inversely proportional to the sample mRNA content. The GAPDH gene expression normalized the IGF-1 gene expression. The samples were normalized by the mean change in the CT (∆CT) animal control, generating the ∆∆CT.

### 4.7. Western Blotting

The EDL muscles were homogenized at 4 °C for 30 s in extraction buffer (100 mM Trizma, pH 7.5; 10 mM EDTA; 100 mM NaF; 10 mM sodium pyrophosphate; 10 mM sodium orthovanadate; 2 mM phenylmethanesulfonyl fluoride; and 0.01 mg/mL aprotinin), and triton X-100 at 1%. The total protein content was determined using bovine serum albumin as the standard [20,69,70].

Sample proteins (75 μg) diluted in Laemmli buffer and dithiothreitol (1 M) were submitted to electrophoresis on polyacrylamide gels and transferred to nitrocellulose membranes at 120 V for 1 h, as previously described [20,69,70]. Non-specific bands were blocked using a basal solution (10 mM Trizma, pH 7.5; 150 mM NaCl; 0.05% Tween 20) containing 5% skim milk at room temperature for 2 h. After washing (three times, 10 min each), the membranes were incubated with the antibodies (from ECM Biosciences, Versailles, France) diluted in a basal solution containing 3% skim milk at room temperature for 3 h: Akt (1:1000 dilution), phospho-Akt (pAkt) (1:1000 dilution, Ser473), p70S6K (1:1000 dilution phospho-p70S6K (pp70S6K) (1:1000 dilution), S6 (1:1000 dilution), phospho-S6 (pS6) (1:1000 dilution), p4E-BP1 (1:1000 dilution), and phospho-4E-BP1 (p4E-BP1) (1:1000 dilution). After washing as above, the membranes were incubated with the corresponding secondary antibody (1:5000) conjugated to horseradish peroxidase in a basal solution containing 1% skim milk at room temperature for 1 h. The membranes were then rewashed and incubated with the peroxidase substrate and chemiluminescence enhancer solution (ECL Western Blotting System Kit, GE Health Care, Little Chalfont, Buckinghamshire, England) for 1 min and immediately exposed to X-ray films. The films were processed, and the band intensities were quantified via optical densitometry using the ImageJ 1.37 software (Wayne Rasband, NIH, USA, http://rsb.info.nih.gov/ij/, accessed on 22 February 2019). GAPDH protein was used to normalize band densities analysis.

### 4.8. Proteasome Activity Assay

The fluorogenic peptide Suc-Leu-Leu-Val-Tyr-7-amido-4-methylcoumarin was used in the proteasome chymotrypsin-like activity assay [71]. Cytosolic protein (50 µg) was diluted in 200 µL of 10 mM MOPS, pH 7.4, containing 25 µM LLVY-MCA, 2.5 µM ATP, and 5.0 mM Mg^2+^. The fluorescent product formation rates were based on measurements recorded at specific excitation (350 nm) and emission (440 nm) wavelengths. The peptidase activity assay was conducted in the absence or presence of 20 µM epoxomicin (a proteasome-specific inhibitor) to confirm the assay’s specificity. In the presence of the inhibitor, no significant proteasome activity was detected.

### 4.9. Statistical Analysis

The results were analyzed using two-way ANOVA followed by the Bonferroni post-test using Prism GraphPad, Version: 5.0 (GraphPad Software Inc., San Diego, CA, USA) and considered statistically significant at *p* < 0.05. Histological analysis was performed using the Anderson–Darling Normality Test. The muscle fiber CSA results were not normally distributed; therefore, differences were considered significant when no overlap existed with the 95% confidence interval of the median (95% CI).

## 5. Conclusions

**Figure 8:**
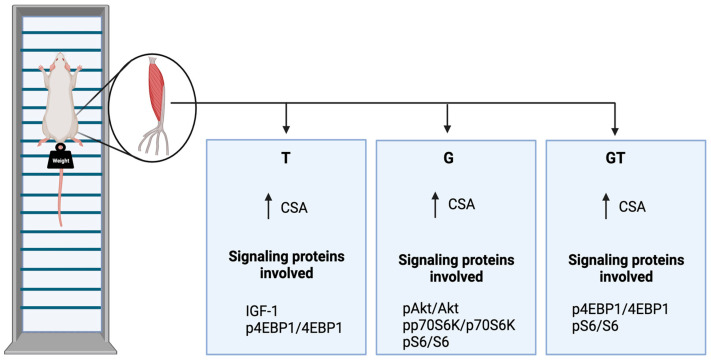
Summary of the findings. Control, no exercise (C); exercise, hypertrophy resistance training exercise protocol (T); no exercise supplemented with glutamine (G); and exercise and supplemented with glutamine (GT). The increase in muscle CSAs in the three experimental groups was mediated via upregulated phosphorylation of Akt, 4E-BP1, p70S6k, and S6 in G and 4E-BP1 in T rats. Proteasome activity was decreased in trained rats, favoring a further increase in the muscle fibers CSA. Abbreviations: CSA, cross-sectional area; IGF1, Insulin-like growth factor 1; phosphorylated-Akt, protein kinase B; phosphorylated-S6, ribosomal protein S6; phosphorylated-4E-BP1, eukaryotic initiation factor 4E-binding protein 1.

Five weeks of GS and/or RET induced muscle hypertrophy, as indicated by the increased CSA of the EDL muscle fibers (Figure 8). The increase in muscle CSA was mediated via the upregulated phosphorylation of Akt, 4E-BP1, p70S6k, and S6 in G and 4E-BP1 in T rats. We detected decreased proteasome activity in the EDL muscles from T animals, favoring a further increase in the EDL muscle CSA (Figure 8). Thus, the 5-week RET in rats provided an applicable experimental protocol for investigating the underlying molecular pathways leading to increased CSA. GS caused similar effects to RET; however, GS did not promote an additive effect when combined with climbing-based exercise.

## Figures and Tables

**Table 1:** Sequences of the primers and annealing temperatures for the real-time PCR analysis of the IGF1 and GAPDH genes.

Gene	Primer Sense (Forward)	Primer Antisense (Reverse)
IGF1	5′-AAGCCTACAAAGTCAGCTCG- 3′	5′-GGTCTTGTTTCCTGCACTT-3′
GAPDH	5′-GATGGGTGTGAACCACGAGAAA-3′	5′-ACGGATACATTGGGGGTAGGA-3′

Abbreviations: IGF1, insulin-like growth factor 1; GAPDH, glyceraldehyde 3-phosphate dehydrogenase.

## Data Availability

The data that support the findings of this study are available in the Supplementary Data.

## Supplementary Materials

The following supporting information can be downloaded at: https://www.mdpi.com/article/10.3390/nu15224711/s1. The Supplementary Materials contain the raw data for RT-PCR, Western blotting analysis, and the original blots.
