# Supplementary material for: Changes in Skeletal Muscle Protein Metabolism Signaling Induced by Glutamine Supplementation and Exercise
Source: Nutrients. 2023 Nov 7;15(22):4711. doi: 10.3390/nu15224711 (PMC10674901; doi:10.3390/nu15224711)
Supplement: Supplementary file 1 [file nutrients-15-04711-s001.zip › Supplemental data 1.pdf]

nutrients

MDPI

S1- Individual data and mean ± SEM related to the concentration of glutamine **(μmol/g of wet weight)** in the EDL muscle that was shown in Figure 1A.

**Groups**							**Mean**	**±SEM**
Control	1.072	1.690	6.974	2.574	3.740		3.210	1.043
Trained	6.678	1.994	5.624	7.090	4.970		5.271	0.901
Glutamine	9.122	5.473	5.952	5.729	7.513	6.668	6.743#	0.563
Glutamine Trained	1.938	0.152	3.871	2.585	3.996	9.493	3.672	1.299

#P= 0.0447 versus Control (Control, no exercise).

S2- Individual data and mean ± SEM related to the concentration of glutamate **(μmol/g of wet weight)** in the EDL muscle that was shown in Figure 1B.

**Groups**							**Mean**	**±SEM**
Control	10.430	8.127	7.034	9.185	7.600	6.083	8.077	0.634
Trained	4.277	6.924	13.031	10.562	3.301	6.417	7.419	1.522
Glutamine	10.414	8.762	10.370	7.771	8.735	8.654	9.118	0.430
Glutamine Trained	14.300	17.435	6.751	6.899	15.716		12.220*	2.258

*P=0.0422 versus Glutamine (supplemented with glutamine).

S3- Individual data and mean ± SEM related to mRNA expression **(arbitrary units)** of IGF-1 (ΔΔCT) in the EDL muscle that was shown in Figure 2.

**Groups**							**Mean**	**±SEM**
Control	0.365	0.730	1.170	0.981	0.931	1.780	0.835	0.137
Trained	1.363	2.261	1.259	2.465	1.401	0.845	1.599	0.256
Glutamine	0.994	1.436	2.080	0.802	0.777		1.218	0.246
Glutamine Trained	1.081	1.312	1.768	1.471	0.640		1.254	0.190

S4- Individual data and mean ± SEM **(uF/mg prot/min)** related to the activity of the 26S proteasome in the EDL muscle that was shown in Figure 7.

**Groups**							**Mean**	**±SEM**
Control	5833.6	6013.5	7551.3	6550.5	6038.8		6397.6	312.1
Trained	4638.3	4775.6	4911.8	4543.1	4276.6		4629.1	107.9
Glutamine	5318.2	5104.0	6662.3	5401.3	5621.7		5621.5	352.5
Glutamine Trained	4953.6	3273.7	4032.1	6391.9	4086.4		4086.5	485.6

S5- Individual data and mean ± SEM **(arbitrary units)** related to total Akt expression in the EDL muscle that was shown in Figure 3A.

**Groups**										**Mean**	**±SEM**
Control	0.75	0.91	1.34	1.22	0.78	0.56	0.69	2.03	0.71	1.00	0.15
Trained	0.69	0.73	1.29	1.22	0.78	0.51	0.81	0.89	1.36	0.92	0.10
Glutamine	0.73	0.68	1.23	1.05	0.47	0.85	1.45	1.01	0.70	0.91	0.10
Glutamine Trained	0.65	0.81	1.03	0.51	0.86	0.86	1.14	0.65		0.81	0.07

S6- Individual data and mean ± SEM **(arbitrary units)** related to phospho-Akt expression in the EDL muscle that was shown in Figure 3A.

**Groups**										**Mean**	**±SEM**
Control	0.90	1.06	1.04	0.63	1.37	*1.73*	0.84	0.86	0.57	0.91	0.09
Trained	0.74	1.03	1.00	*3.51*	1.79	1.27	1.14	1.19	0.86	1.13	0.11
Glutamine	0.92	0.93	1.48	2.22	2.94	*0.82*	1.05	1.00	1.53	1.50	0.30
Glutamine Trained	0.84	0.40	0.71	*3.93*	0.85	*1.25*	1.04	1.08		0.82	0.10

S7- Individual data and mean ± SEM **(arbitrary units)** related to p70S6K expression in the EDL muscle that was shown in Figure 3B.

**Groups**										**Mean**	**±SEM**
Control	0.33	1.72	0.95	1.00	1.00	0.69	0.50	2.04	0.77	1.00	0.18
Trained	0.87	1.17	0.94	0.62	1.44	0.79	0.59	0.73	1.20	0.93	0.10
Glutamine	1.15	0.82	0.65	1.61	0.59	0.52	1.21	1.11	0.99	0.96	0.12
Glutamine Trained	1.22	0.33	1.54	0.76	1.02	0.48	0.90	0.82		0.88	0.14

S8- Individual data and mean ± SEM (arbitrary units) related to phospho p70S6K expression in the EDL muscle that was shown in Figure 3B.

**Groups**										**Mean**	**±SEM**
Control	1.44	0.53	1.03	0.39	1.61	0.84	1.44	1.16	0.56	1.00	0.15
Trained	1.16	0.63	1.30	2.85	*4.67*	2.66	1.87	2.09	1.38	1.74	0.27
Glutamine	1.33	*0.3*	1.80	4.6	3.70	2.19	1.96	1.39		2.43	0.48
Glutamine Trained	1.20	0.97	1.18	0.81	1.87	*2.8*	1.85	1.21		1.30	0.15

S9- Individual data and mean ± SEM **(arbitrary units)** related to 4E-BP-1 expression in the EDL muscle that was shown in Figure 4A.

**Groups**										**Mean**	**±SEM**
Control	1.02	0.97	1.01	0.90	1.10	0.57	0.66	1.83	0.94	1.00	0.12
Trained	1.28	1.03	1.03	0.95	1.22	0.76	0.66	0.92	1.42	1.03	0.08
Glutamine	1.28	0.98	1.02	1.04	0.99	0.77	0.66	1.23	1.07	1.00	0.07
Glutamine Trained	1.13	0.85	0.99	0.91	0.86	0.81	0.96	0.98		0.94	0.04

S10- Individual data and mean ± SEM **(arbitrary units**) related to phospho-4E-BP-1 expression in the EDL muscle that was shown in Figure 4A.

**Groups**										**Mean**	**±SEM**
Control	0.26	0.72	*2.0*	0.88	1.12	*1.9*	0.92	1.09	0.02	0.72	0.16
Trained	1.79	1.95	2.02	1.31	1.03	1.45	1.46	1.56	1.36	1.55	0.11
Glutamine	2.10	2.04	2.22	1.31	1.26	1.49	2.89	1.40	0.18	1.65	0.26
Glutamine Trained	1.80	1.42	2.49	1.43	1.31	2.00	1.59	0.36		1.55	0.22

S11- Individual data and mean ± SEM **(arbitrary units)** related to S6 in the EDL muscle that was shown in Figure 4B.

**Groups**										**Mean**	**±SEM**
Control	1.06	1.07	0.87	0.93	1.07	0.39	1.53	1.11	0.96	1	0.10
Trained	1.26	0.97	0.98	0.72	1.17	0.81	0.23	1.70	1.11	1.0	0.13
Glutamine	1.21	0.90	0.84	0.91	1.05	0.57	1.76	0.46	1.50	1.02	0.14
Glutamine Trained	0.96	0.79	0.81	0.96	0.94	0.79	0.33	1.01		0.82	0.08

S12- Individual data and mean ± SEM **(arbitrary units)** related to phospho S6 in the EDL muscle that was shown in Figure 4B.

**Groups**										**Mean**	**±SEM**
Control	0.65	1.35	0.99	0.68	1.32	3.6	0.21	0.09	0.01	0.67	0.19
Trained	1.08	1.35	1.59	0.80	1.19	1.43	0.72	0.38	0.77	1.03	0.13
Glutamine	1.81	1.28	2.21	1.07	2.07	0.81	0.52	0.91	0.0	1.33	0.22
Glutamine Trained	2.29	0.87	0.94	1.58	1.62	0.77	1.30	0.39		1.22	0.21

S13- Individual data and mean ± SEM related to correlation between glutamine content and pAkt, pp70s6k, ppS6and and p4E-BP1 in EDL muscle that was shown in Figure 5.

Correlation	
[ ] glutamine(**μmol/g of wet weight)**	pAkt/Akt
1.071857	0.5717271
1.689591	0.6273903
6.974119	1.37261
2.574214	0.8390242
3.740237	0.8574782
6.677788	1.785968
1.994272	0.7409461
5.624057	1.269518
7.089653	3.51391
4.969769	1.135292
9.121946	2.943125
5.473167	0.9289021
5.952058	0.9961811
5.728602	1.05004
7.512558	2.216612
6.668298	1.480855
1.937868	0.8505196
0.1516113	0.4025652
2.585443	0.8433271
3.995713	1.079599
9.492702	3.937783

Correlation	
[ ] glutamine(**μmol/g of wet weight)**	pp70S6k/p70S6k
1.071857	0.3859021
1.689591	0.5311077
6.974119	1.614098
2.574214	1.438635
3.740237	1.442538
6.677788	2.853678
1.994272	1.872911
5.624057	2.663212
7.089653	4.66921
4.969769	2.089499
9.121946	4.642411
5.473167	1.390897
5.952058	1.956738
5.728602	1.802863
7.512558	3.701925
6.668298	2.185637
1.937868	0.9738358
0.1516113	0.8115995
3.870826	1.176371
2.585443	0.9738358
3.995713	1.200014

Correlation	
[ ] glutamine **(µmol/g of wet weight)**	pS6/S6
1.071857	0.6534495
1.689591	0.6832098
6.974119	1.352748
2.574214	0.9938027
3.740237	1.31679
6.677788	1.428447
1.994272	0.7189625
5.624057	1.594049
7.089653	1.189378
4.969769	1.352877
9.121946	2.206876
5.473167	1.280463
5.952058	1.074896
5.728602	0.9060218
7.512558	2.066415
6.668298	1.813441
1.937868	0.7732537
0.1516113	0.3946728
3.870826	0.9433405
2.585443	0.8679298
3.995713	1.296098

Correlation	
[ ] glutamine **(µmol/g of wet weight)**	p4E-BP1/4EBP
1.071857	0.2626576
1.689591	0.7186592
6.974119	1.972691
2.574214	0.9170692
3.740237	1.088183
6.677788	1.952667
1.994272	1.034601
5.624057	1.462388
7.089653	1.952667
4.969769	1.359782
9.121946	2.889044
5.473167	2.099412
3.751498	1.258162
5.952058	1.487695
5.728602	1.404084
7.512558	2.222065
1.937868	1.422192
0.1516113	0.3579331
3.870826	1.800891
2.585443	1.429933
3.995713	1.59257
