# Supplementary material for: Changes in Skeletal Muscle Protein Metabolism Signaling Induced by Glutamine Supplementation and Exercise
Source: Nutrients. 2023 Nov 7;15(22):4711. doi: 10.3390/nu15224711 (PMC10674901; doi:10.3390/nu15224711)

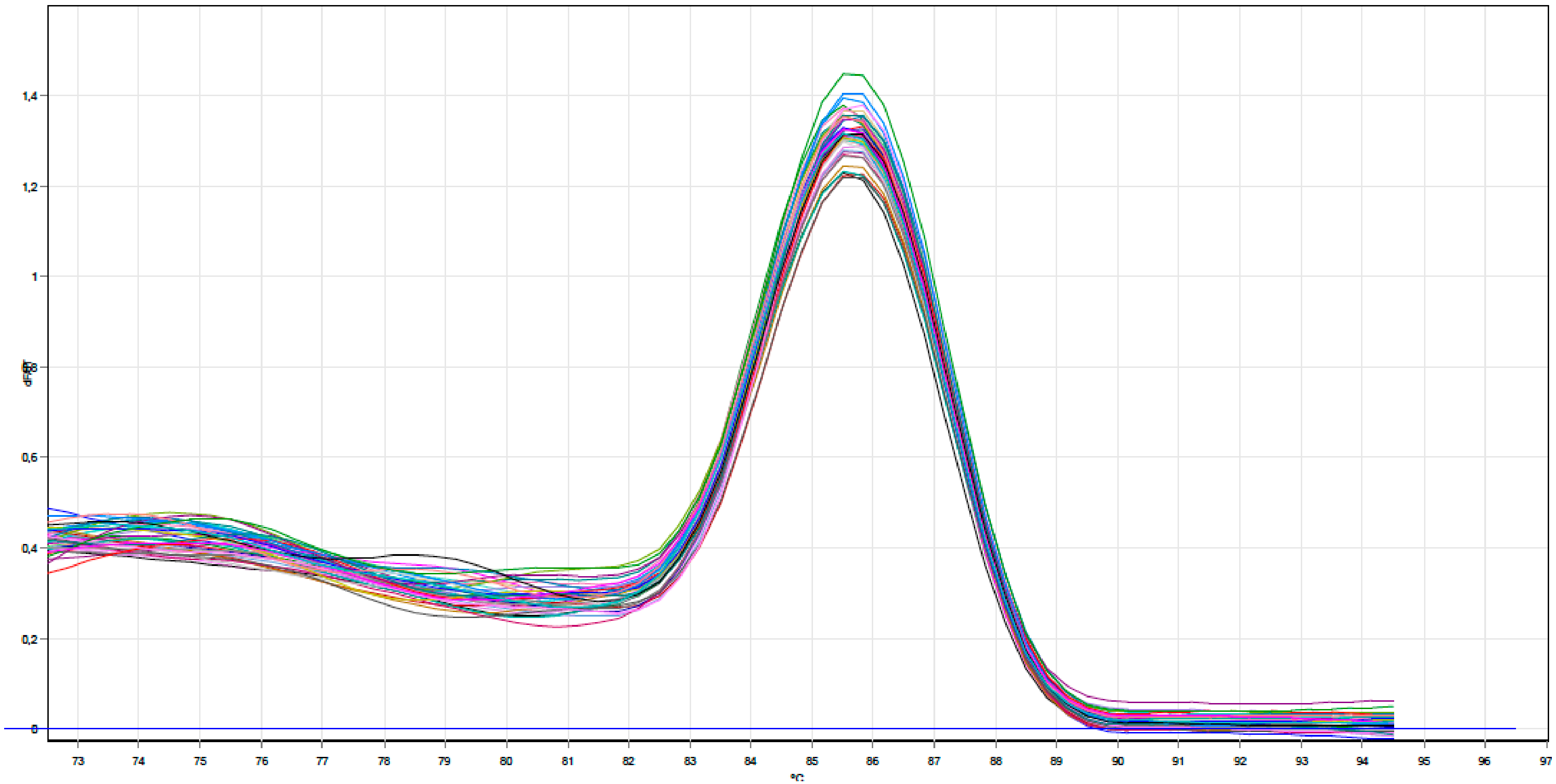

S14: **Melting curve of the GAPDH gene expression.** Melting curve from samples of cDNA from EDL muscle used to real time PCR analysis performed in Figure 2.

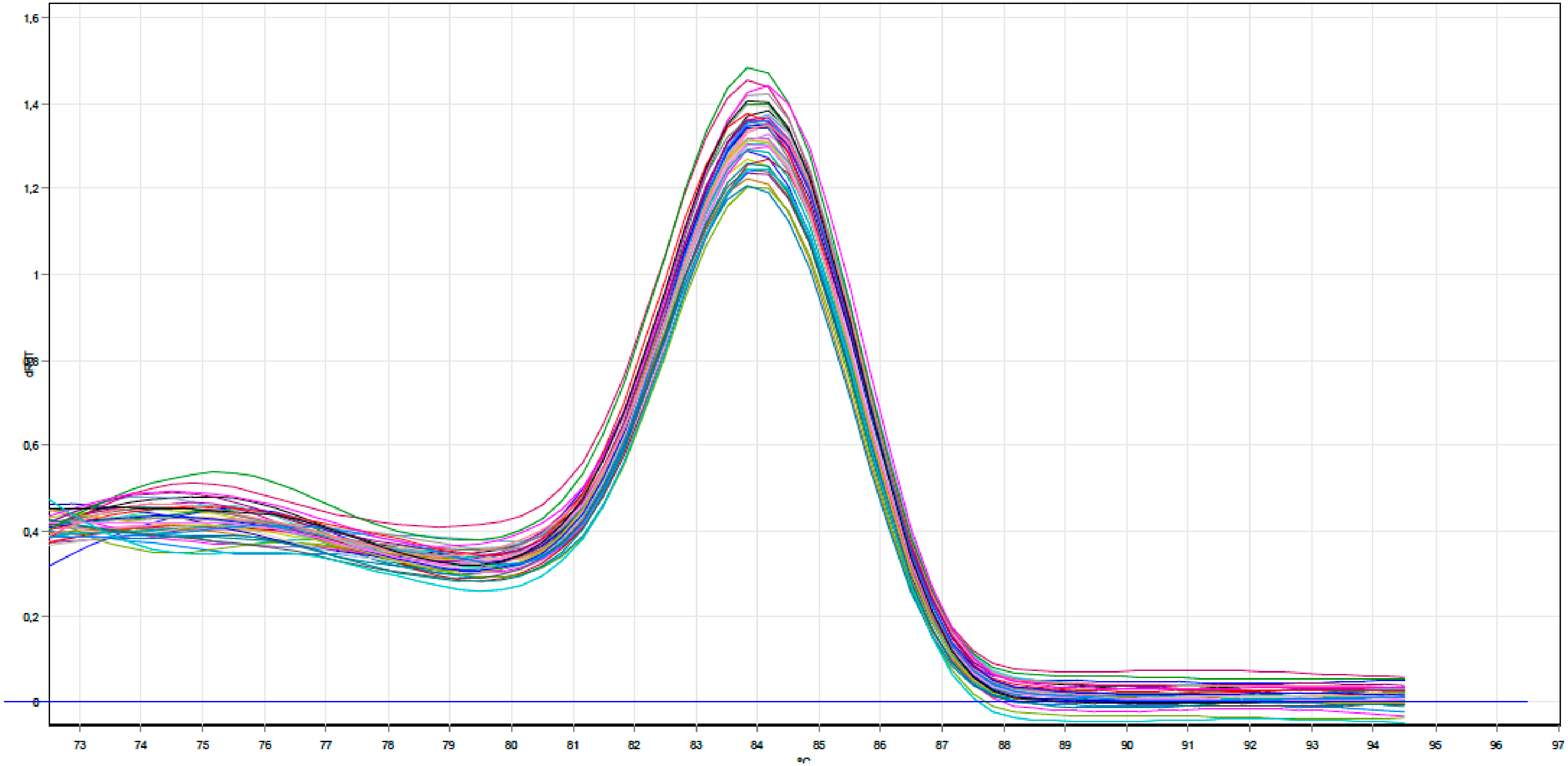

S15: **Melting curve of the IGF-1 gene expression.** Melting curve from samples of cDNA from EDL muscle used to real time PCR analysis performed in Figure 2.

Original results for AKT

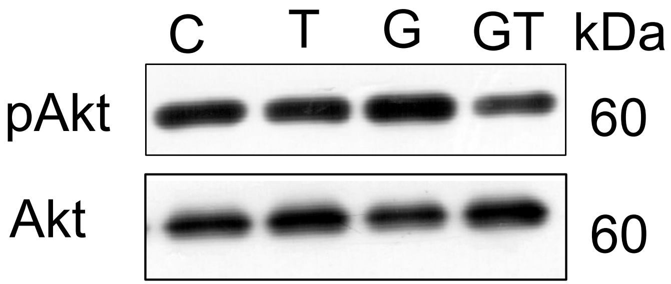

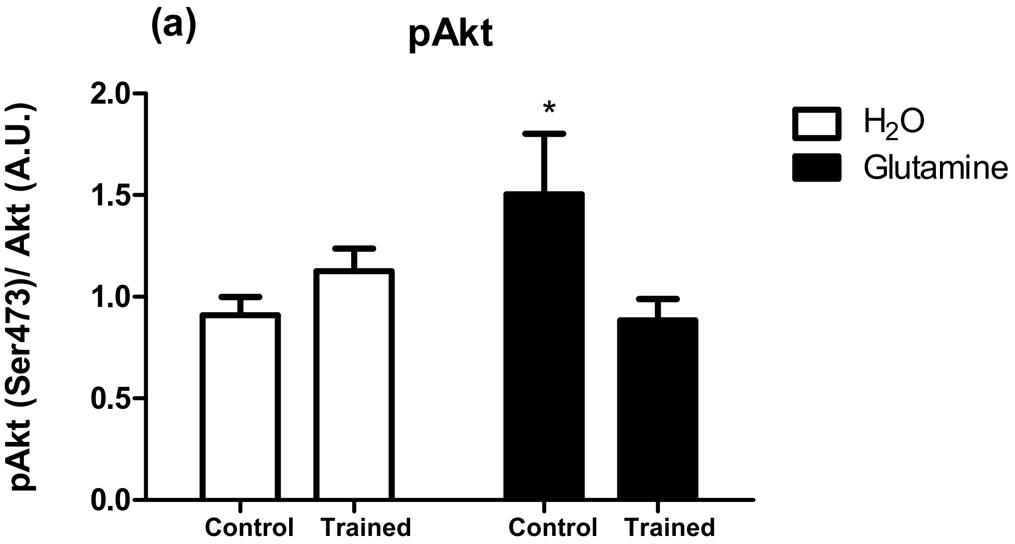

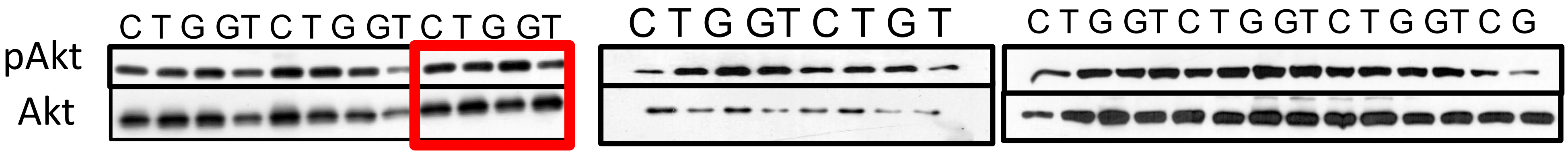

S16: Original data of Akt western-blot. Control, no exercise (C); exercise, hypertrophy resistance training exercise protocol (T); no exercise supplemented with glutamine (G); and exercise and supplemented with glutamine (GT).

Blot 1: pAkt

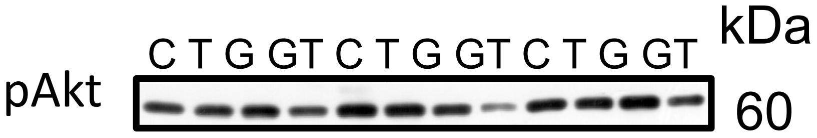

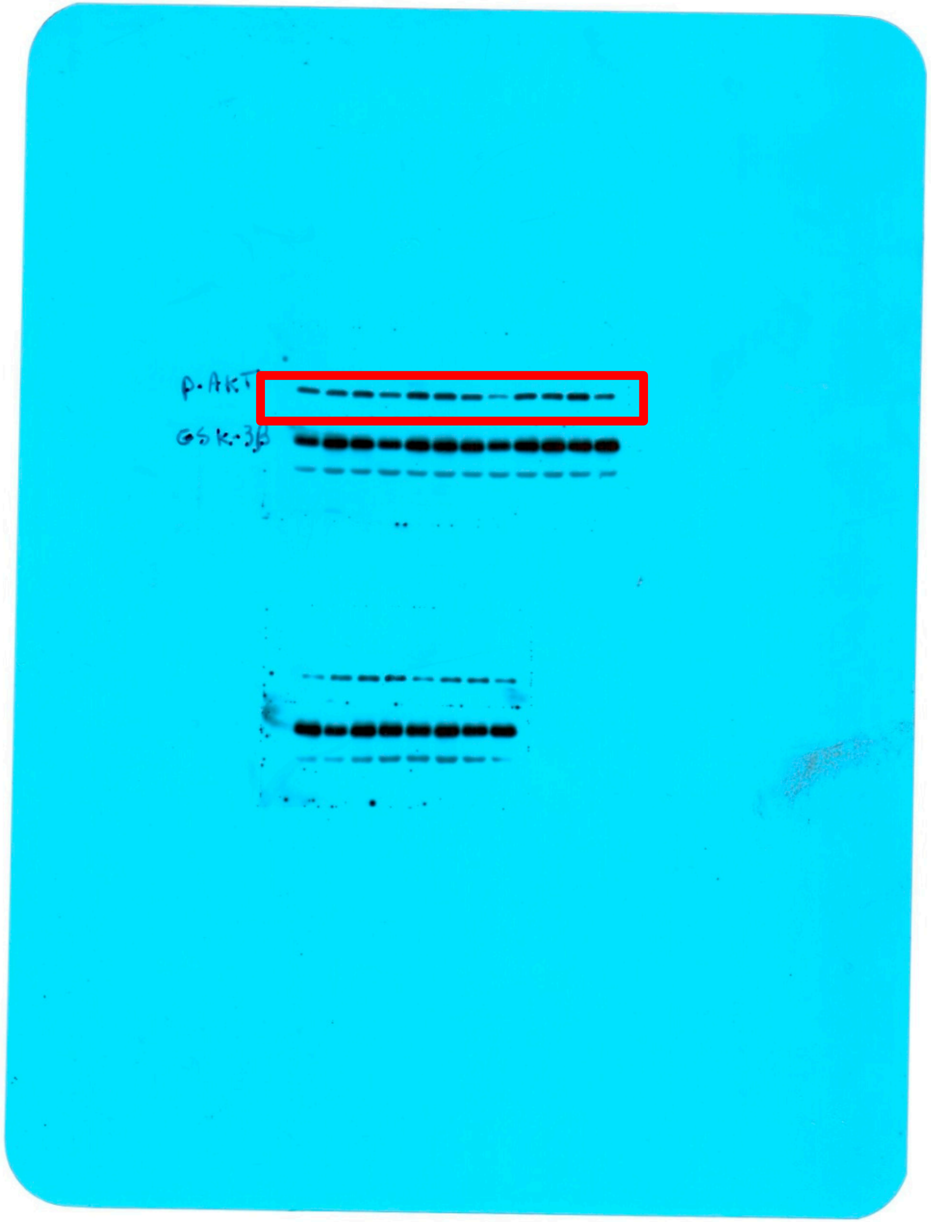

Blot 1: total Akt

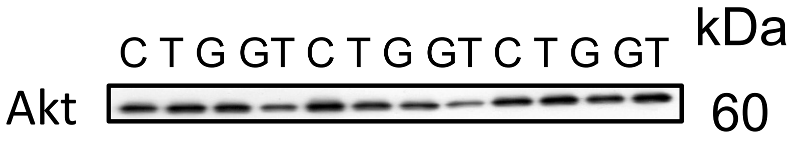

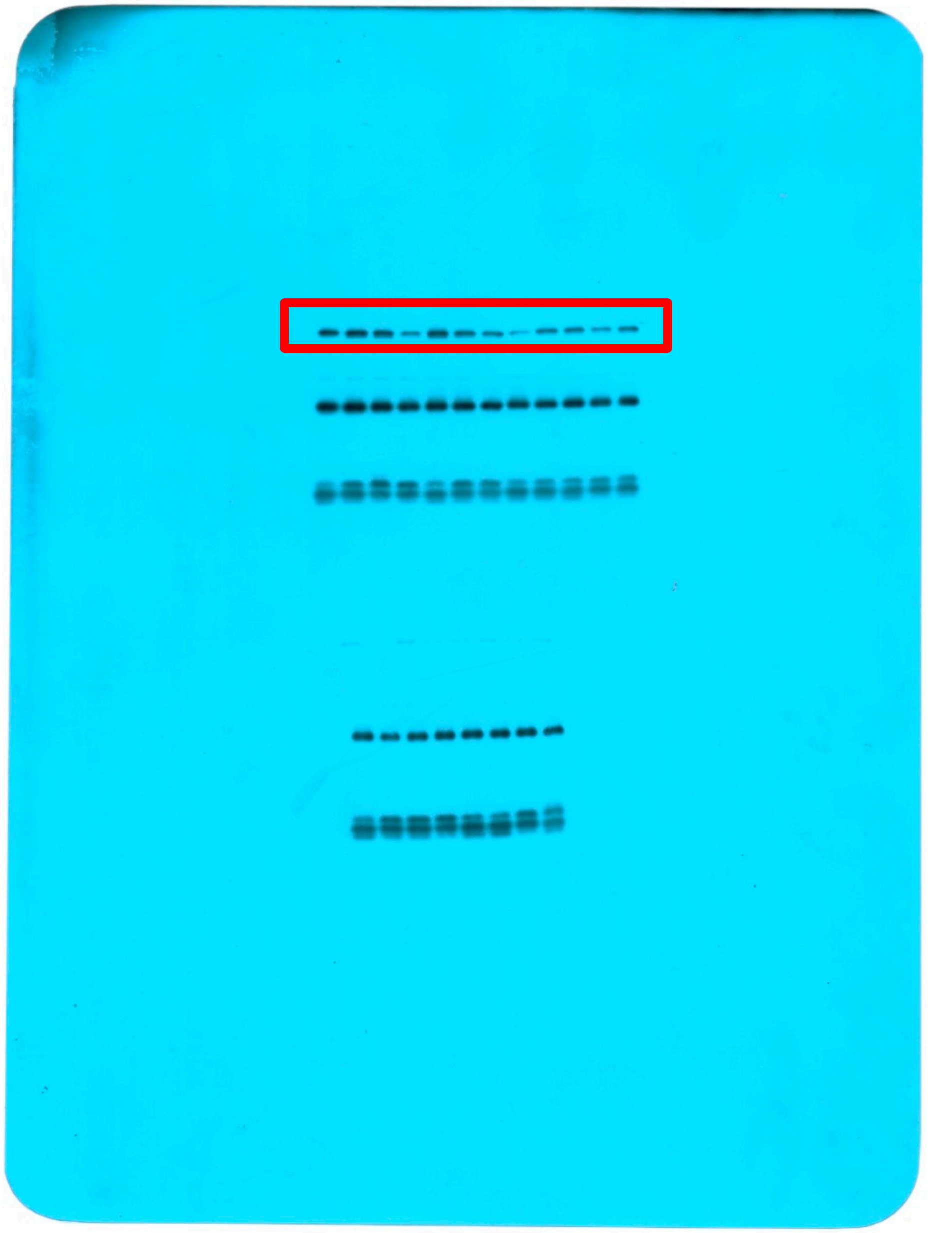

Blot 2: pAkt

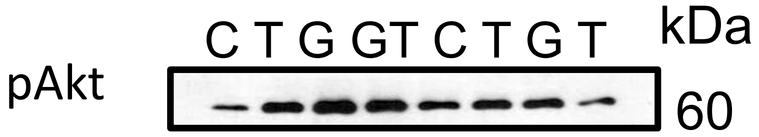

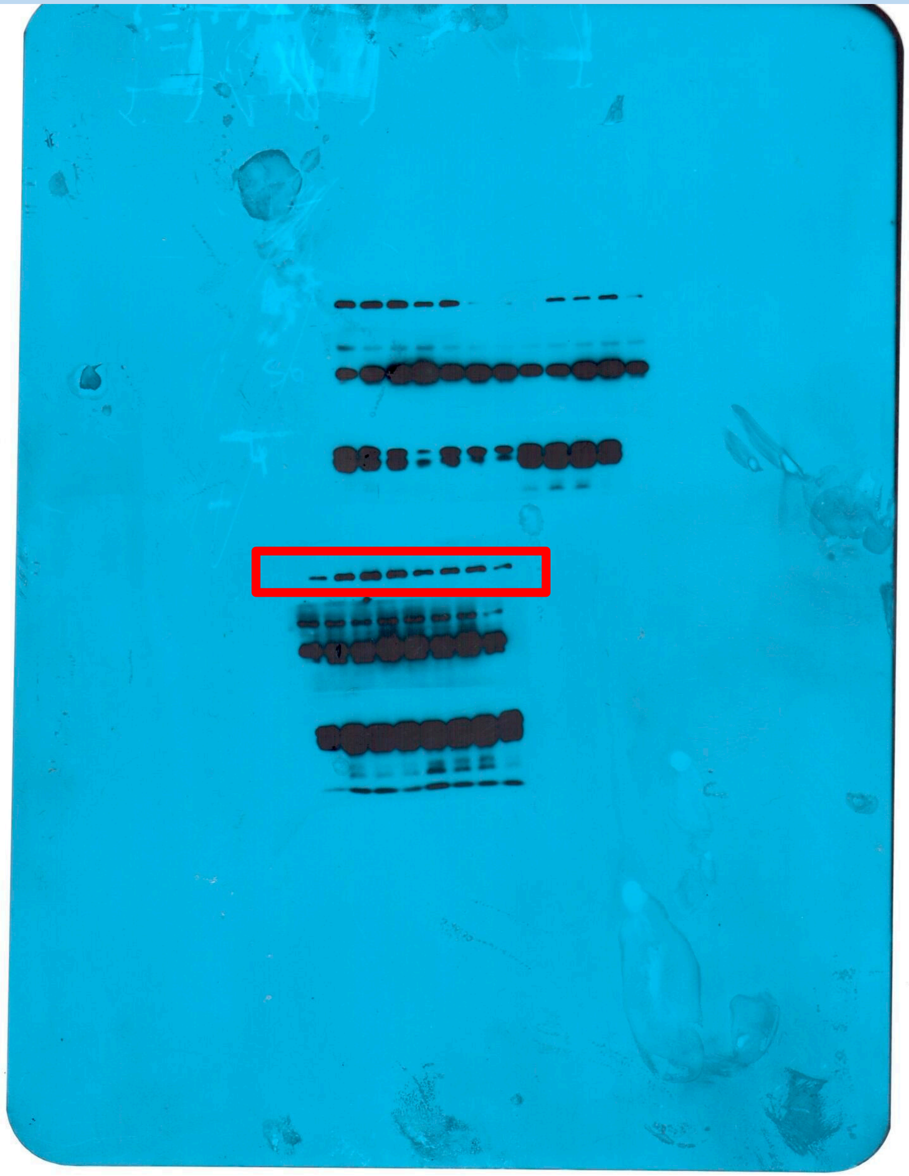

Blot 2: total Akt

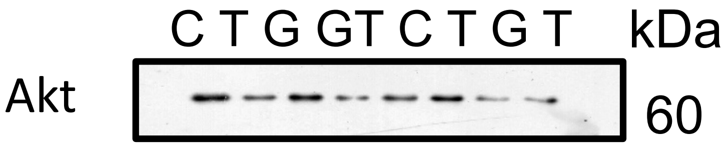

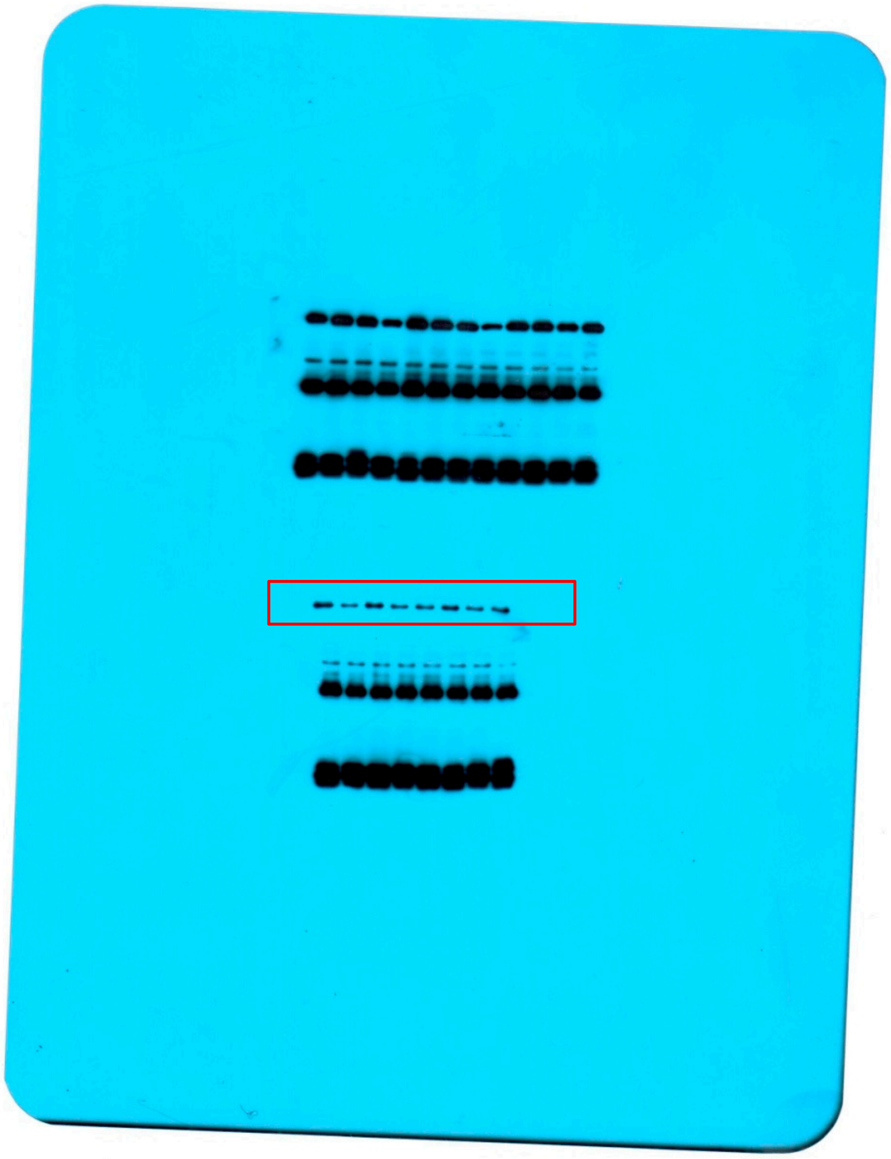

Blot 3: pAkt

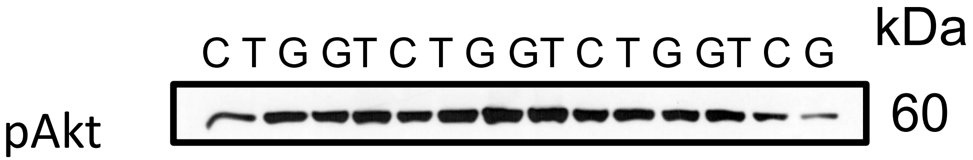

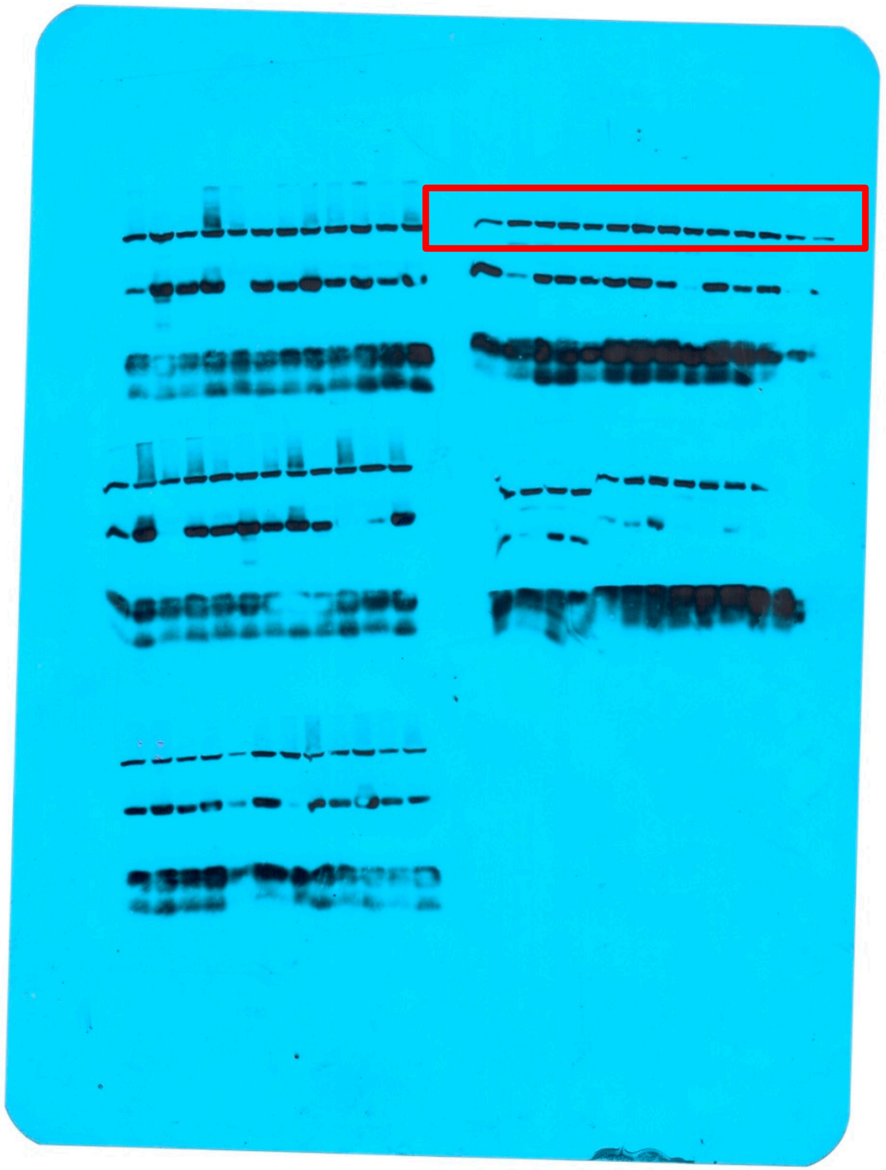

Blot 3: total Akt

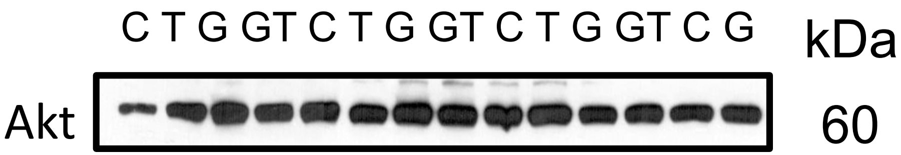

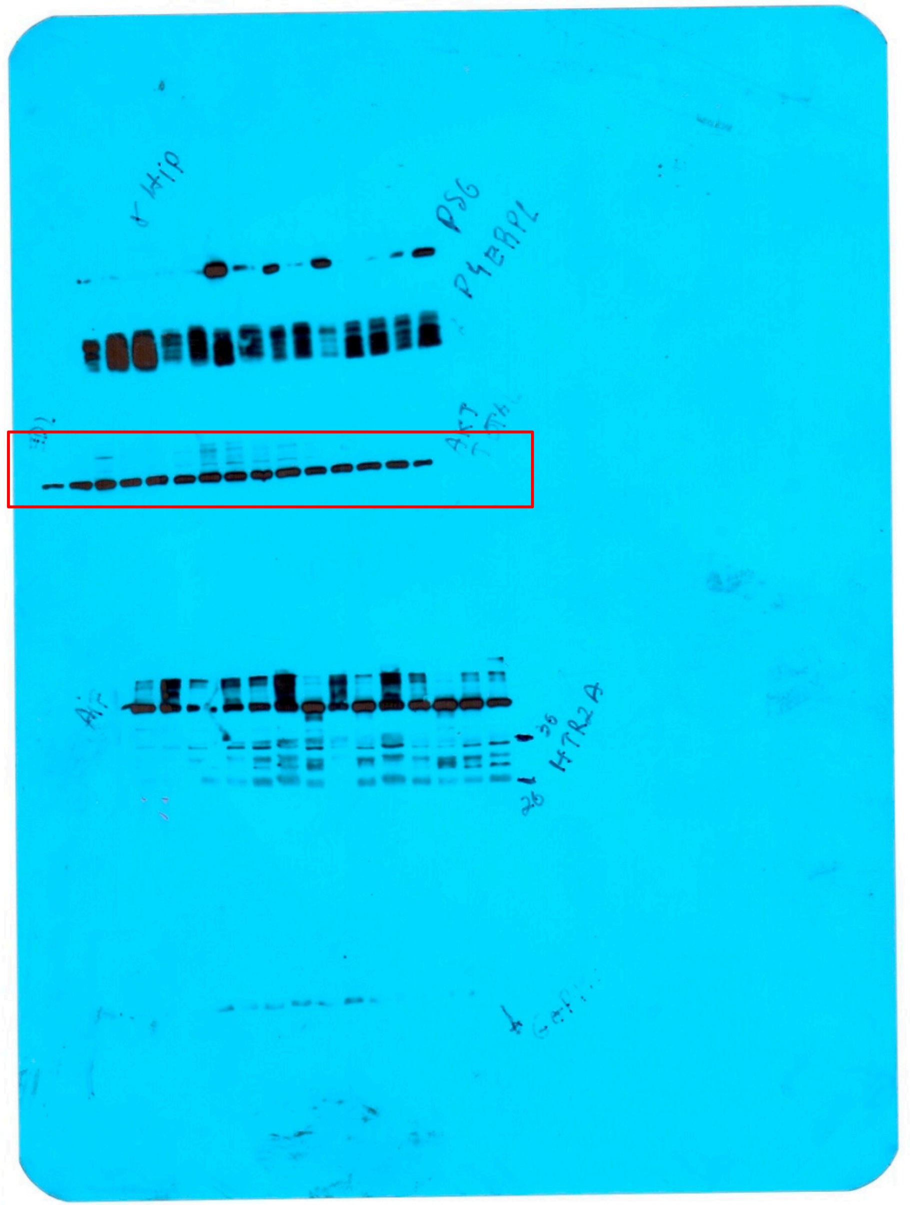

Original results for p70S6K

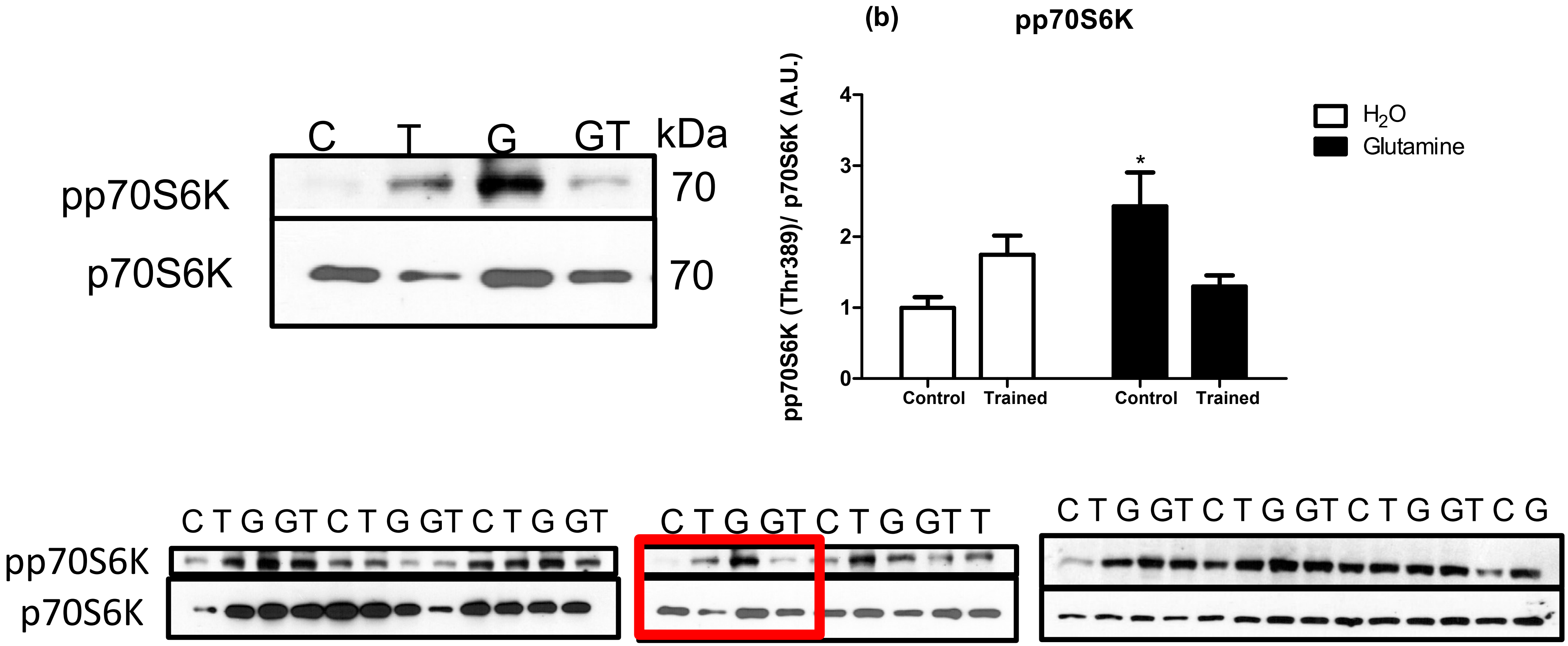

S17: Original data of p70S6K western-blot. Control, no exercise (C); exercise, hypertrophy resistance training exercise protocol (T); no exercise supplemented with glutamine (G); and exercise and supplemented with glutamine (GT).

Blot 1 and 2: p-p70S6K

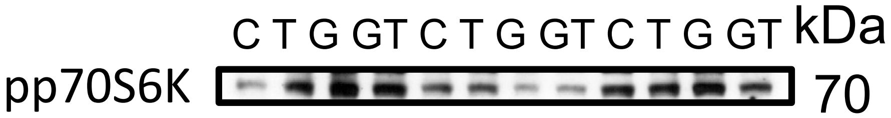

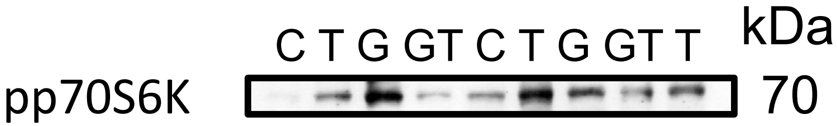

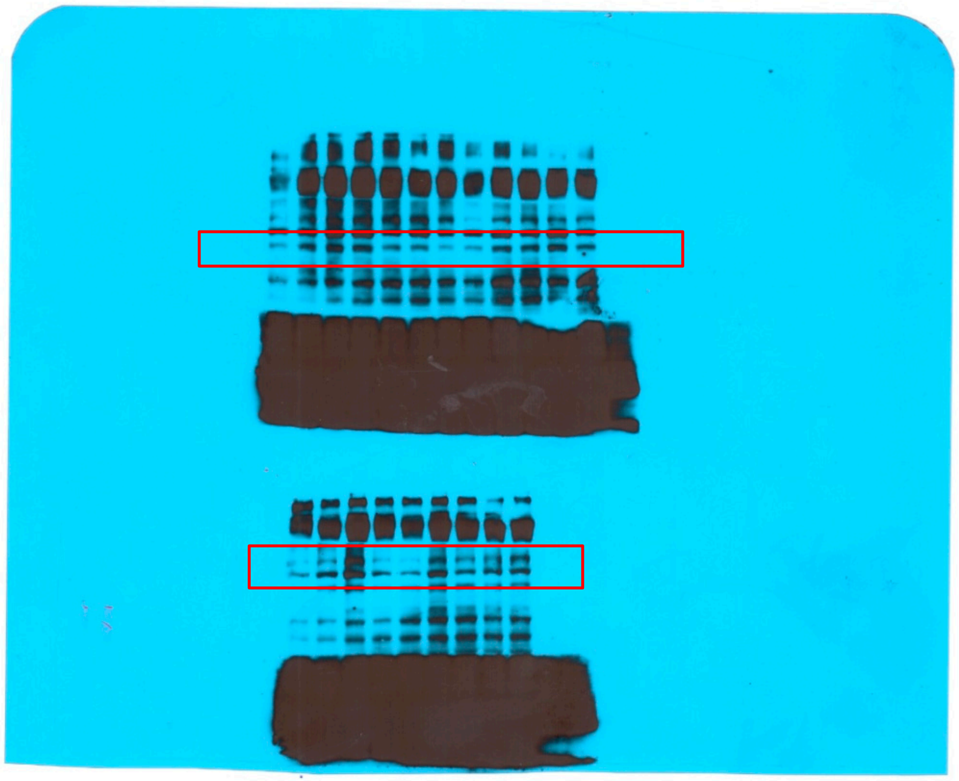

Blot 1 and 2: total p70S6K

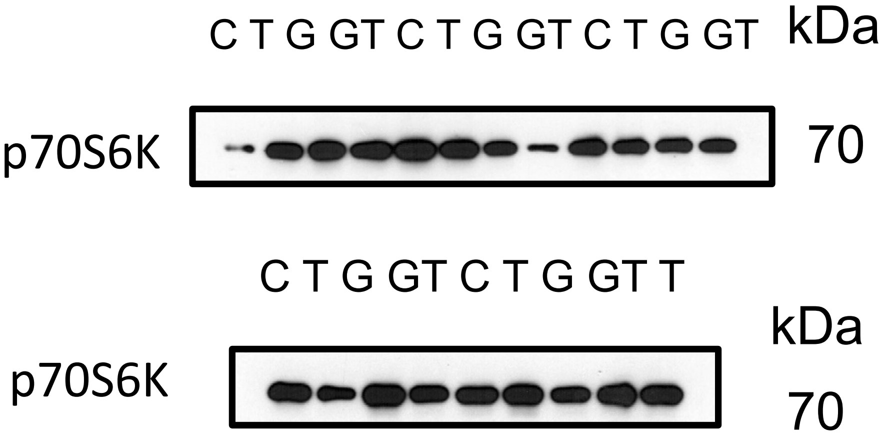

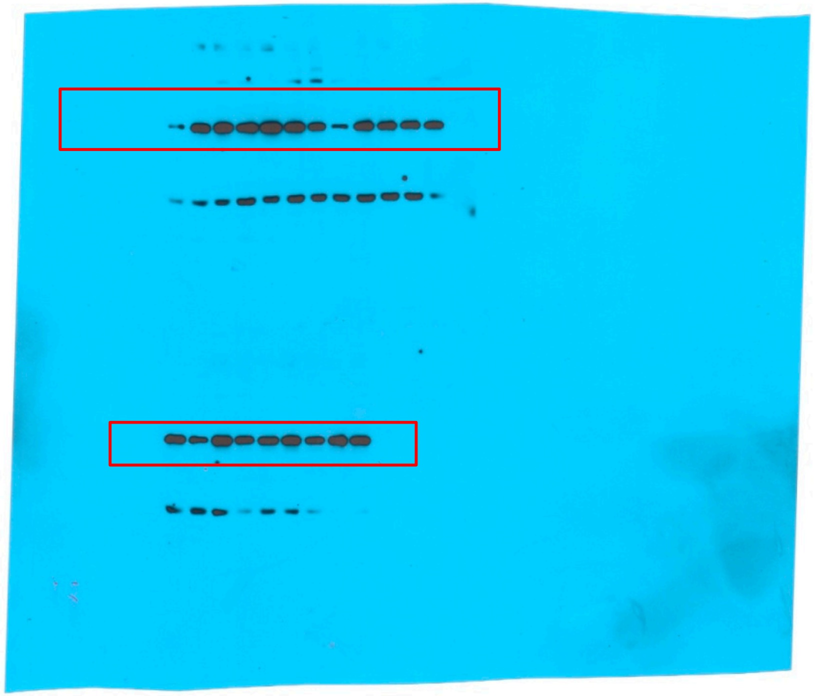

Blot 1 and 2: total p70S6K

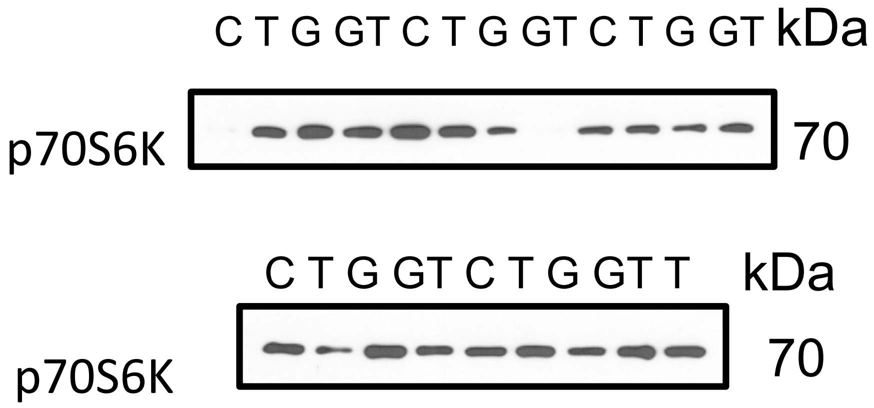

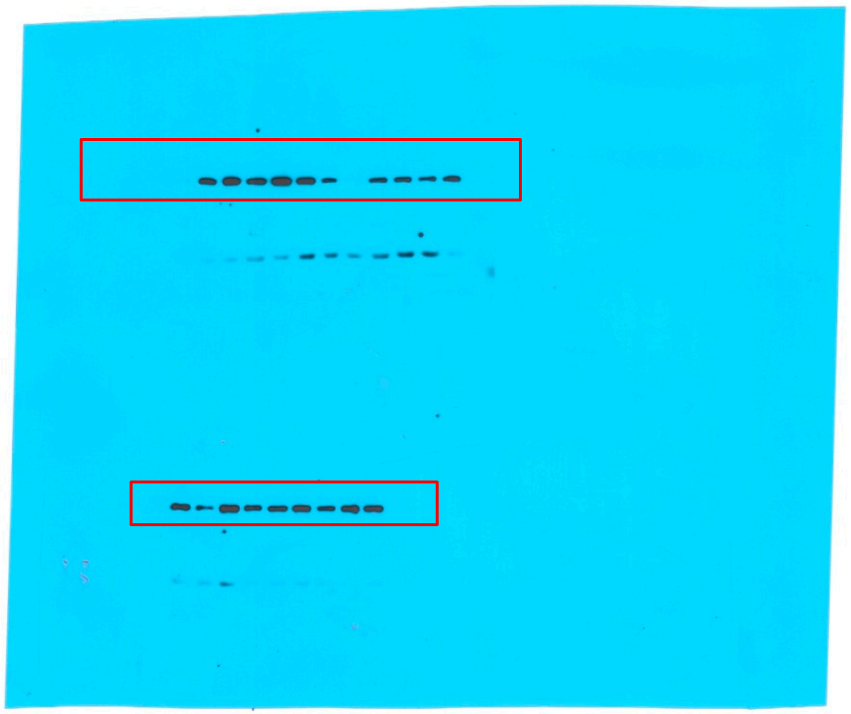

Blot 3: p-p70S6K

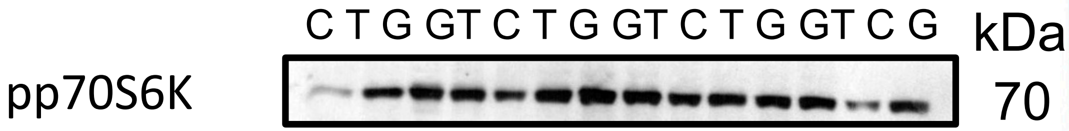

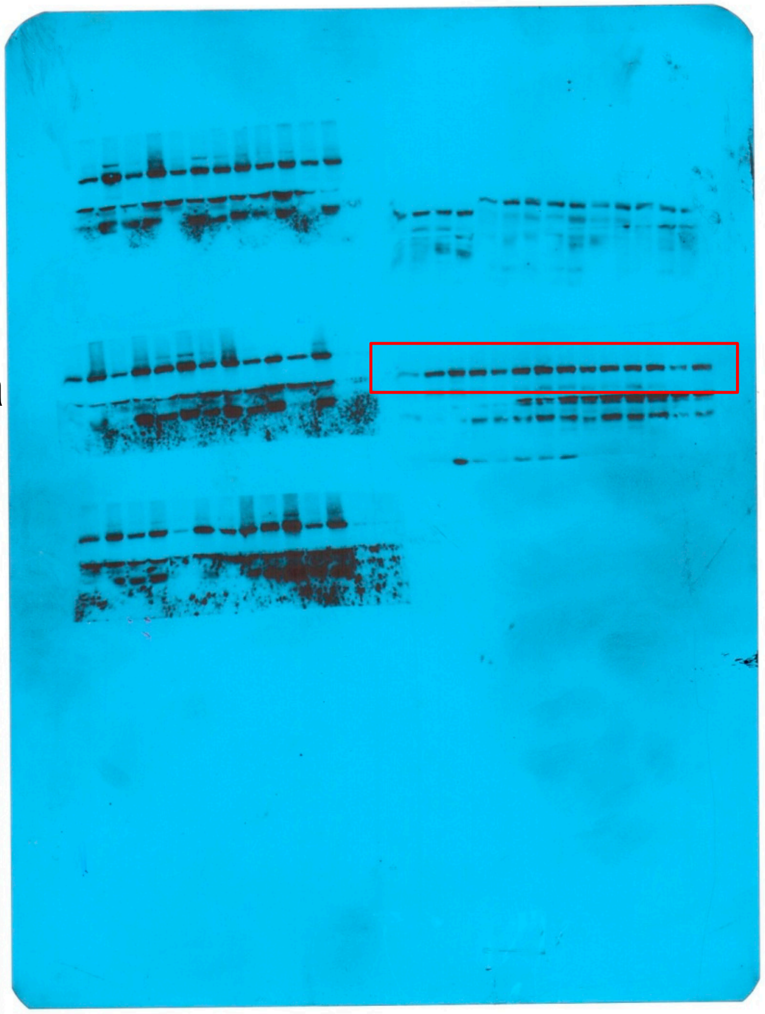

Blot 3: total p70S6K

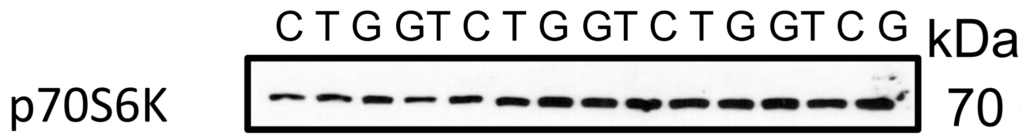

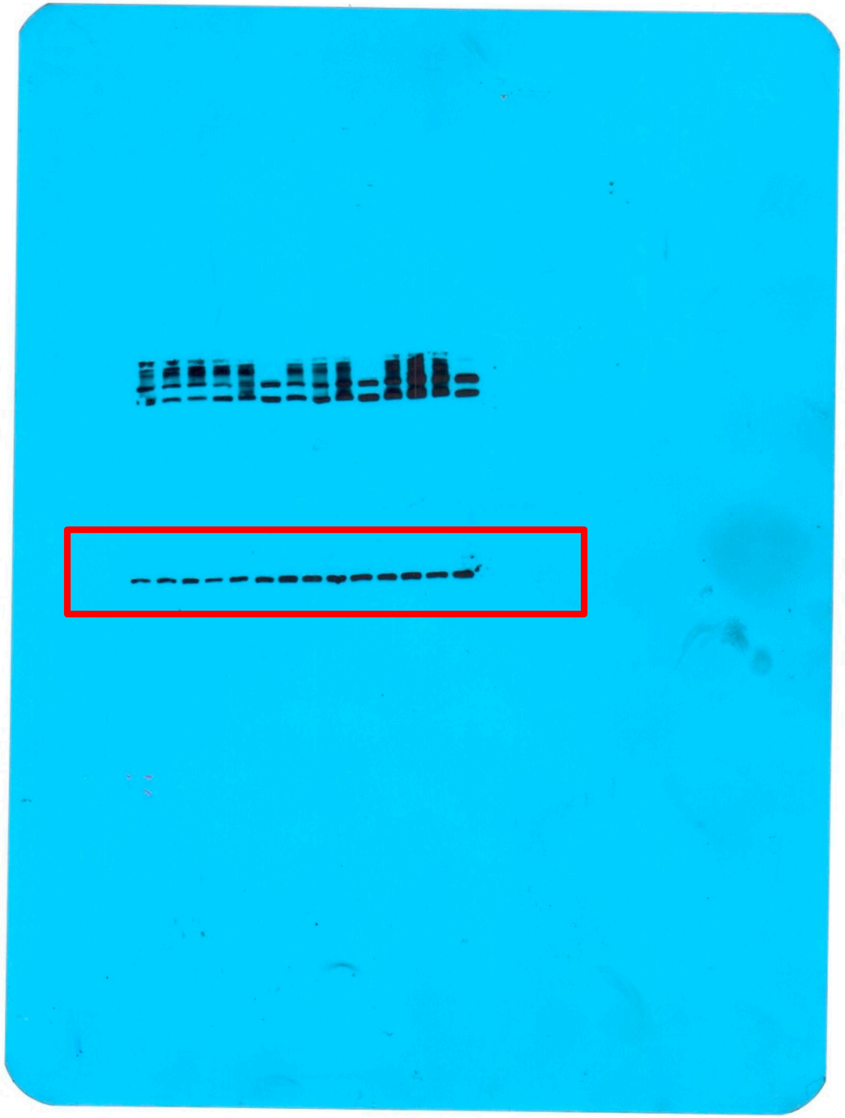

Original results for 4E-BP1

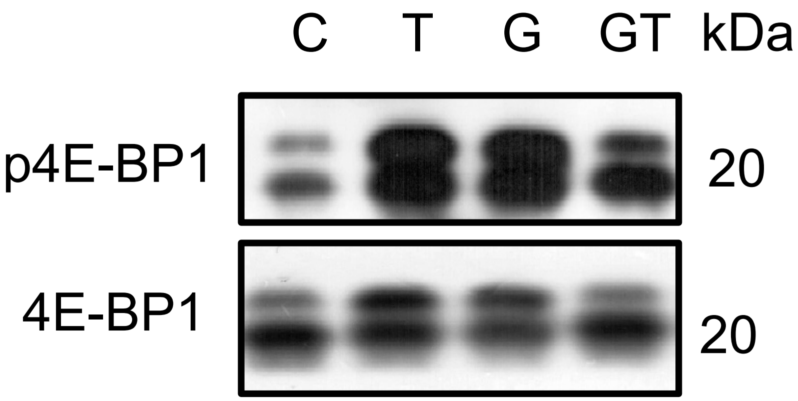

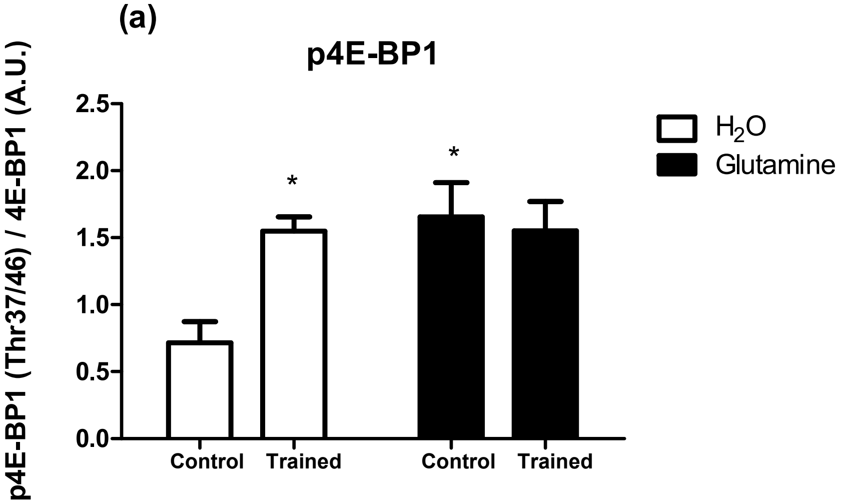

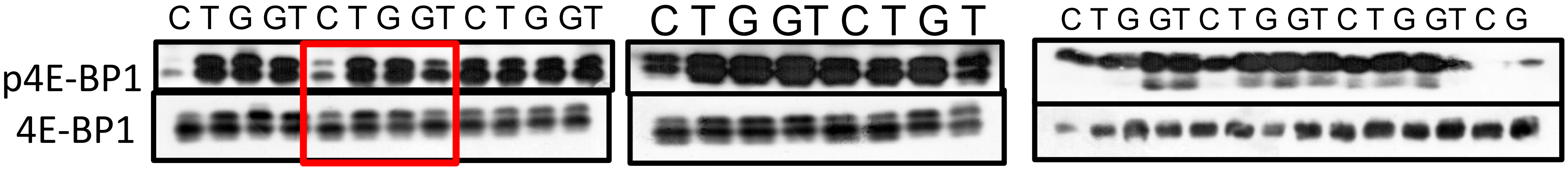

S18: Original data of 4E-BP1 western-blot. Control, no exercise (C); exercise, hypertrophy resistance training exercise protocol (T); no exercise supplemented with glutamine (G); and exercise and supplemented with glutamine (GT).

Blot 1 and 2: p4E-BP1

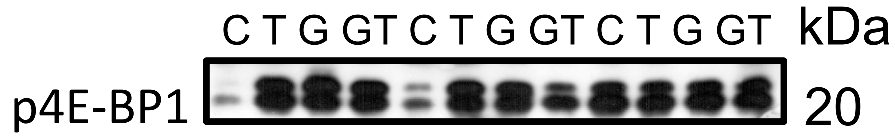

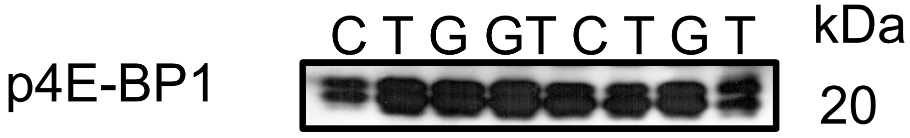

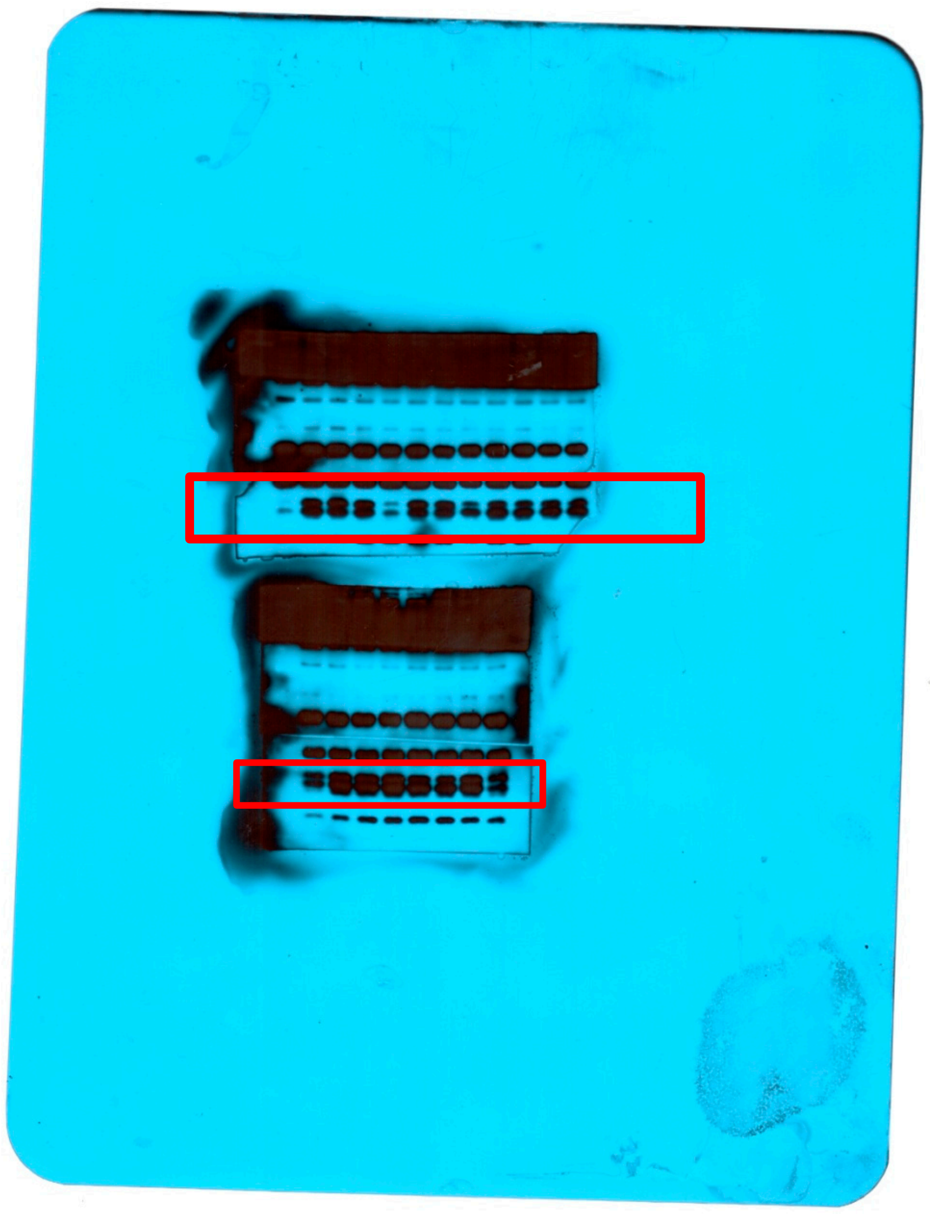

Blot 1 and 2: total 4E-BP1

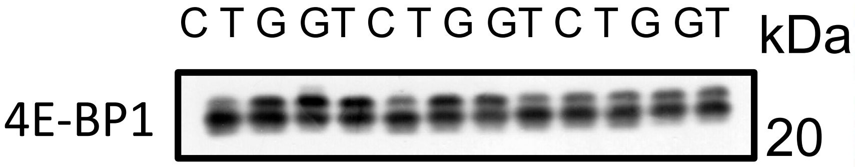

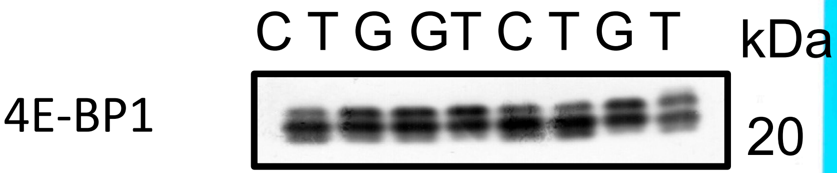

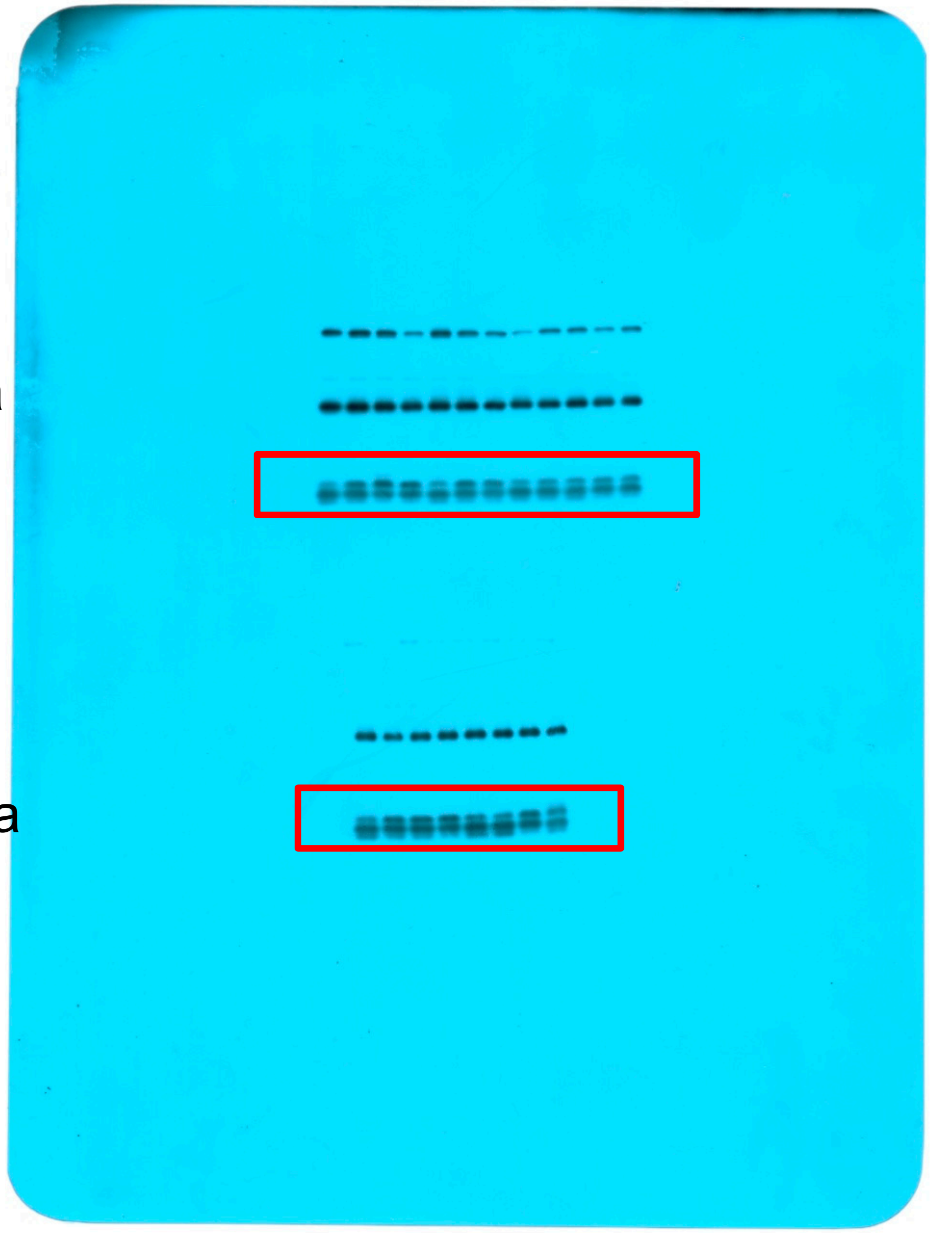

Blot 3: p4E-BP1

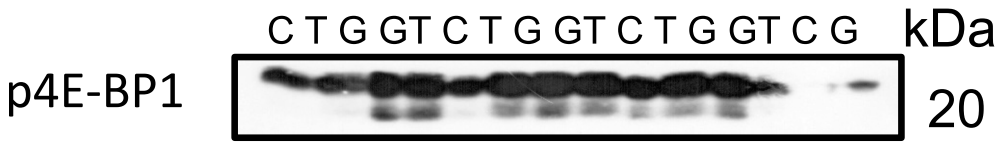

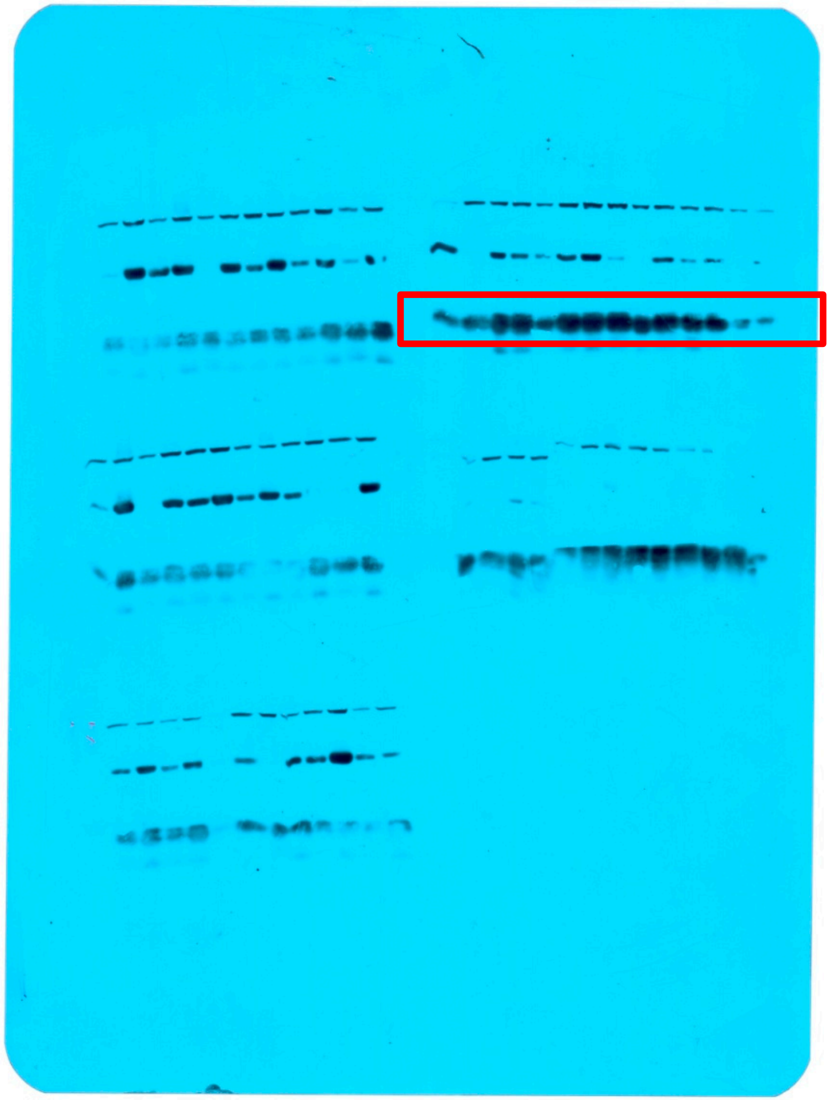

Blot 3: total 4E-BP1

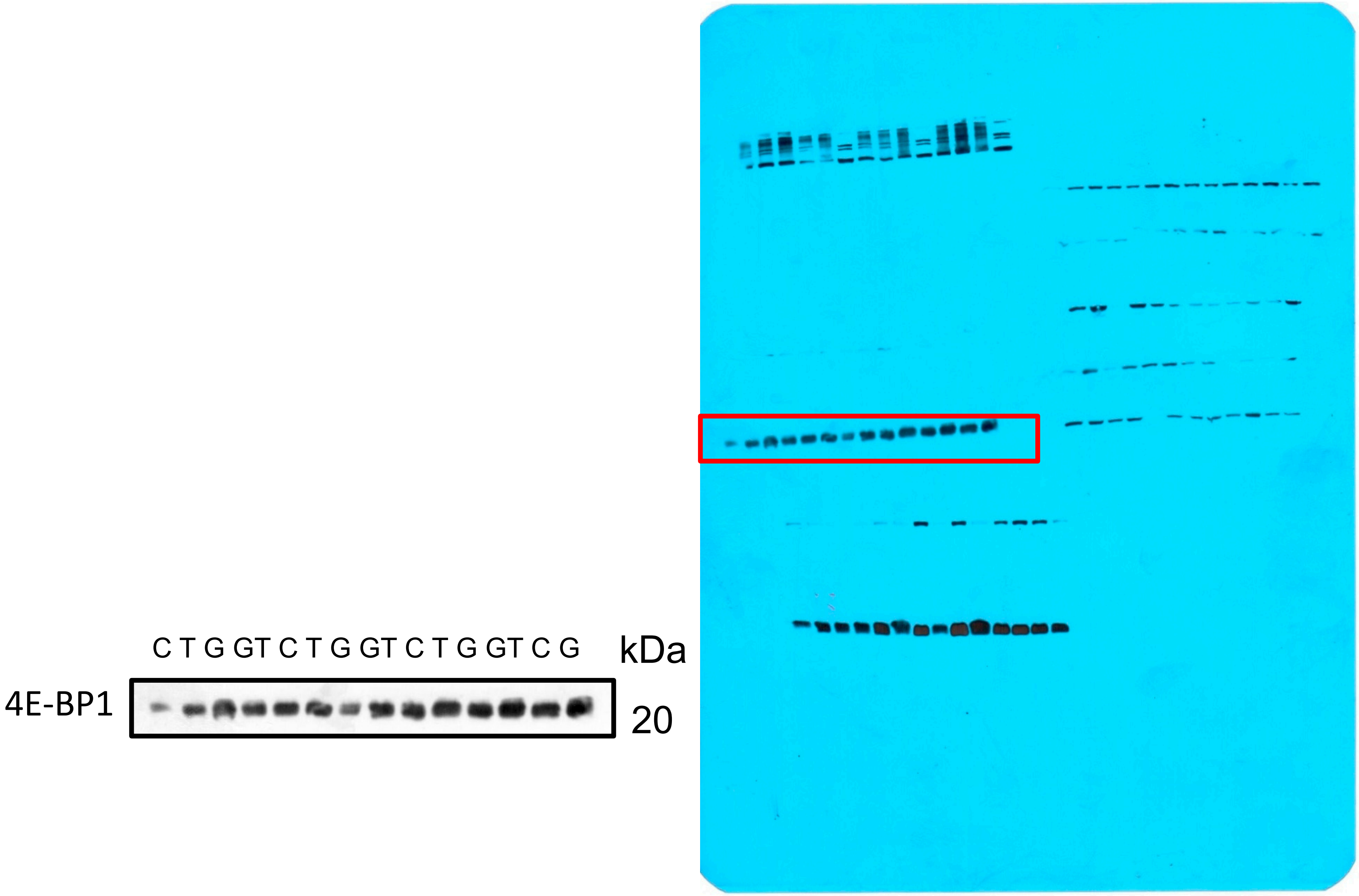

Original results for pS6

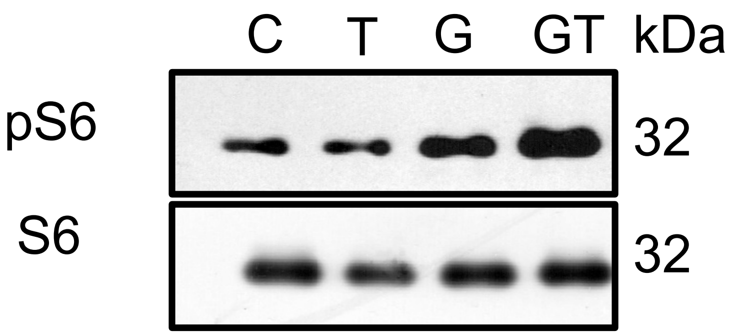

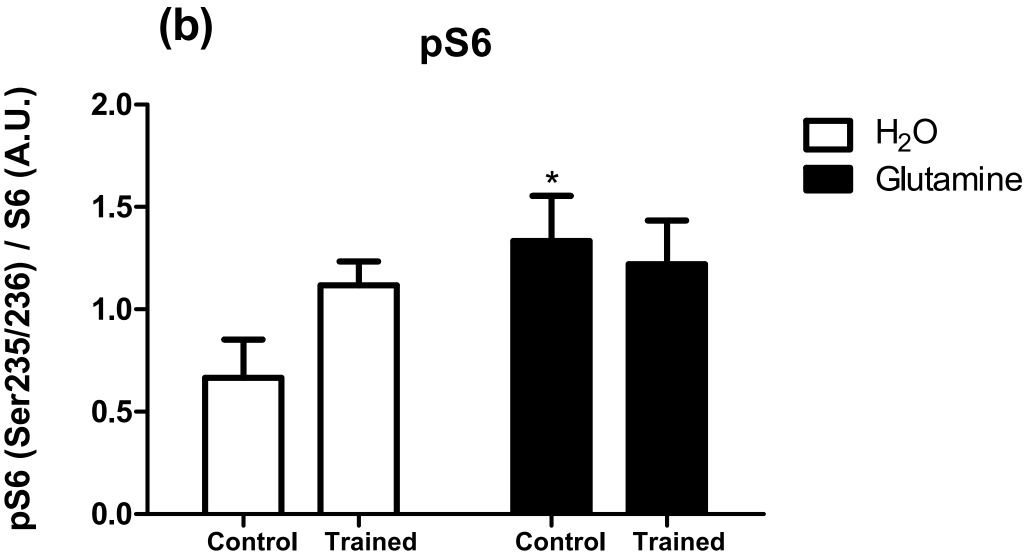

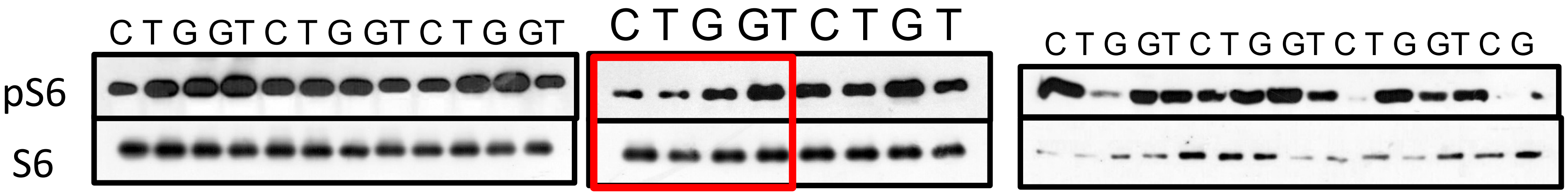

S19: Original data of pS6 western-blot. Control, no exercise (C); exercise, hypertrophy resistance training exercise protocol (T); no exercise supplemented with glutamine (G); and exercise and supplemented with glutamine (GT).

Blot 1 and 2: pS6

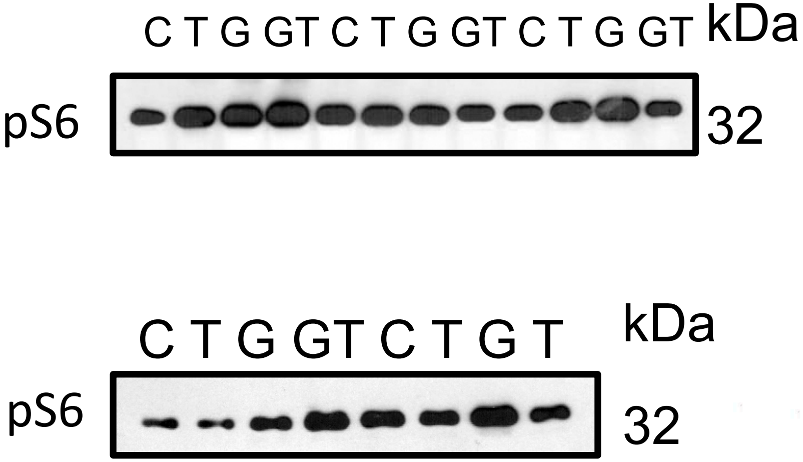

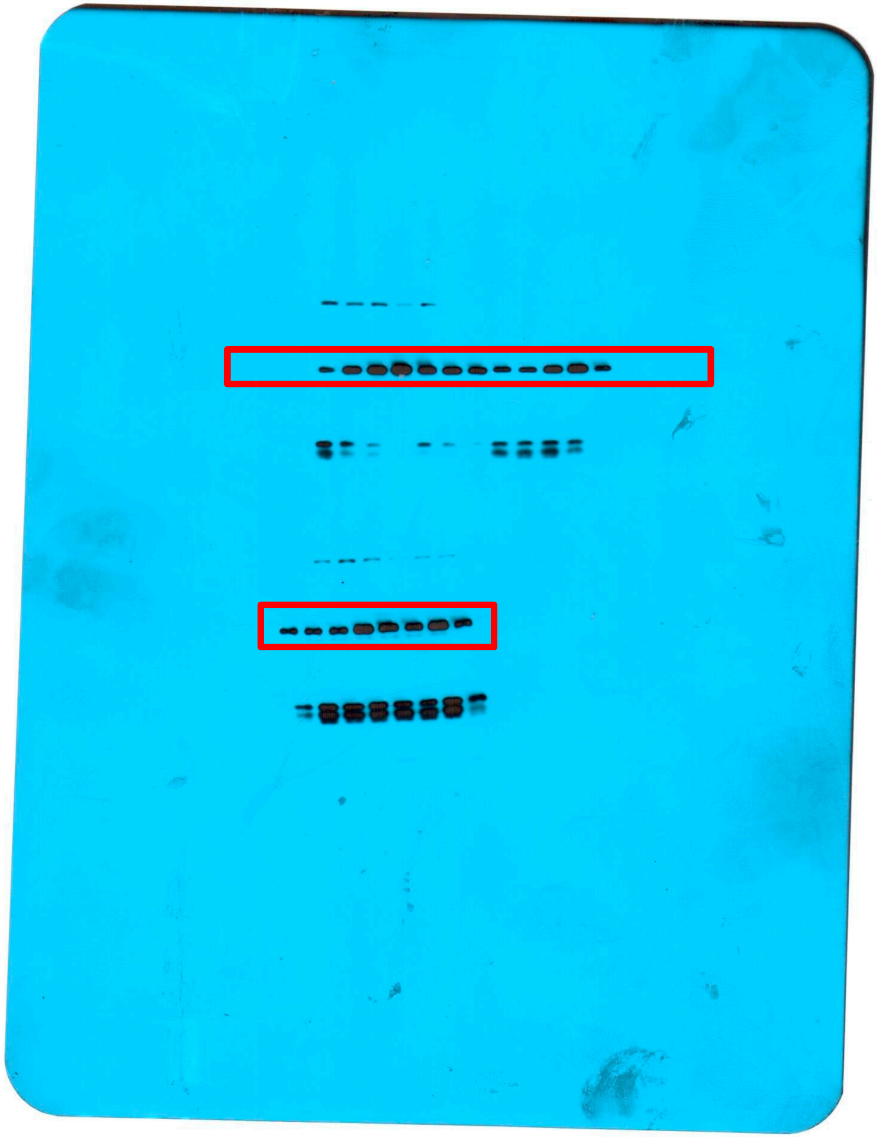

Blot 1 and 2: total S6

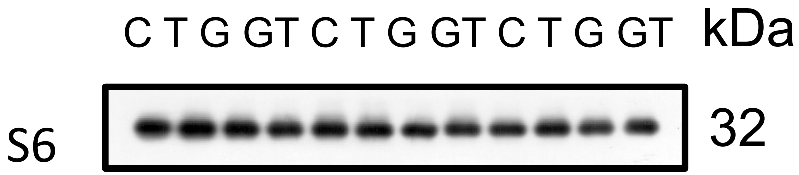

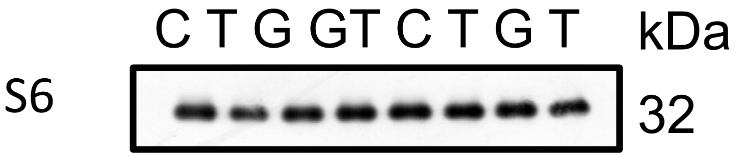

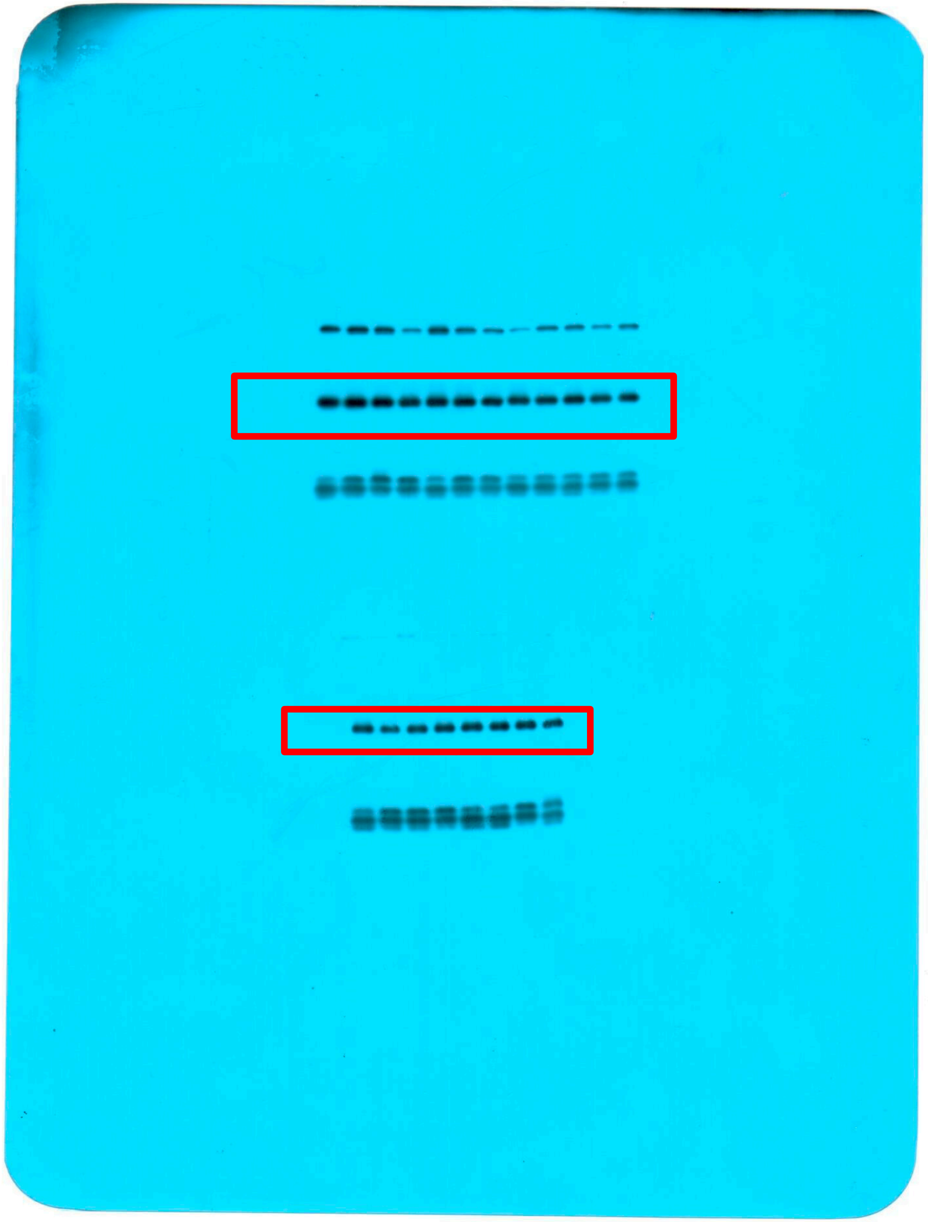

Blot 3: pS6

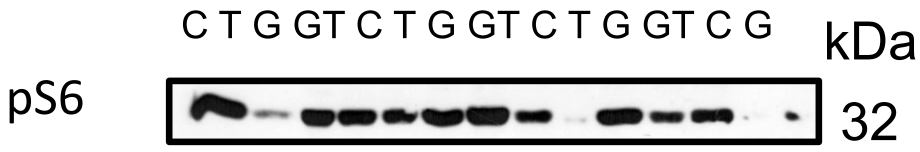

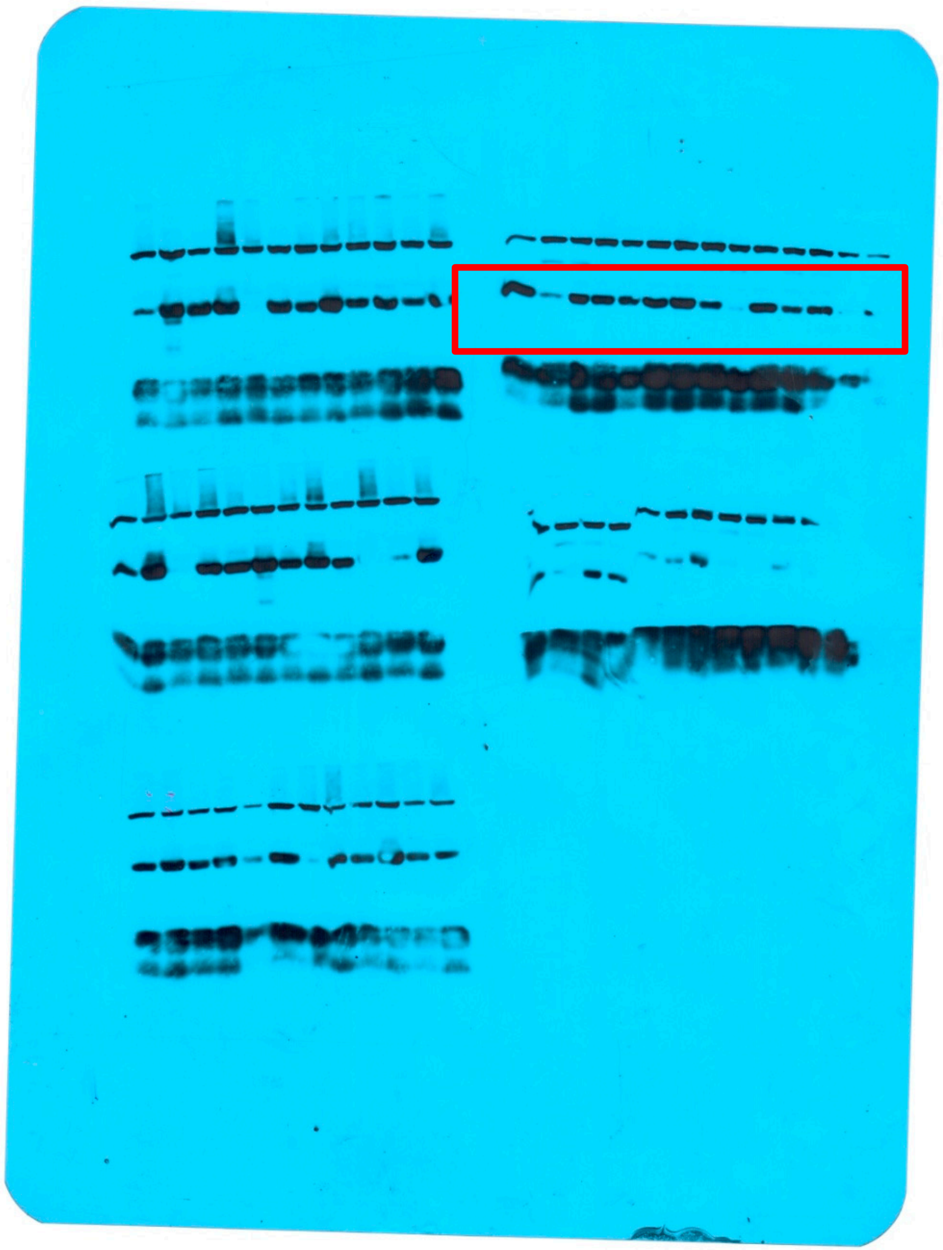

Blot 3: total S6

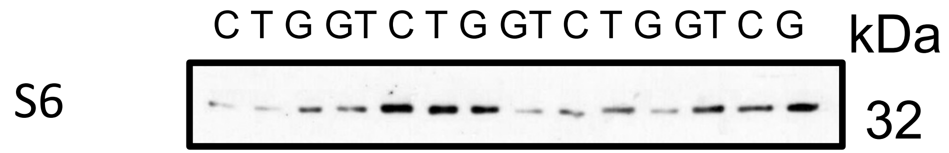

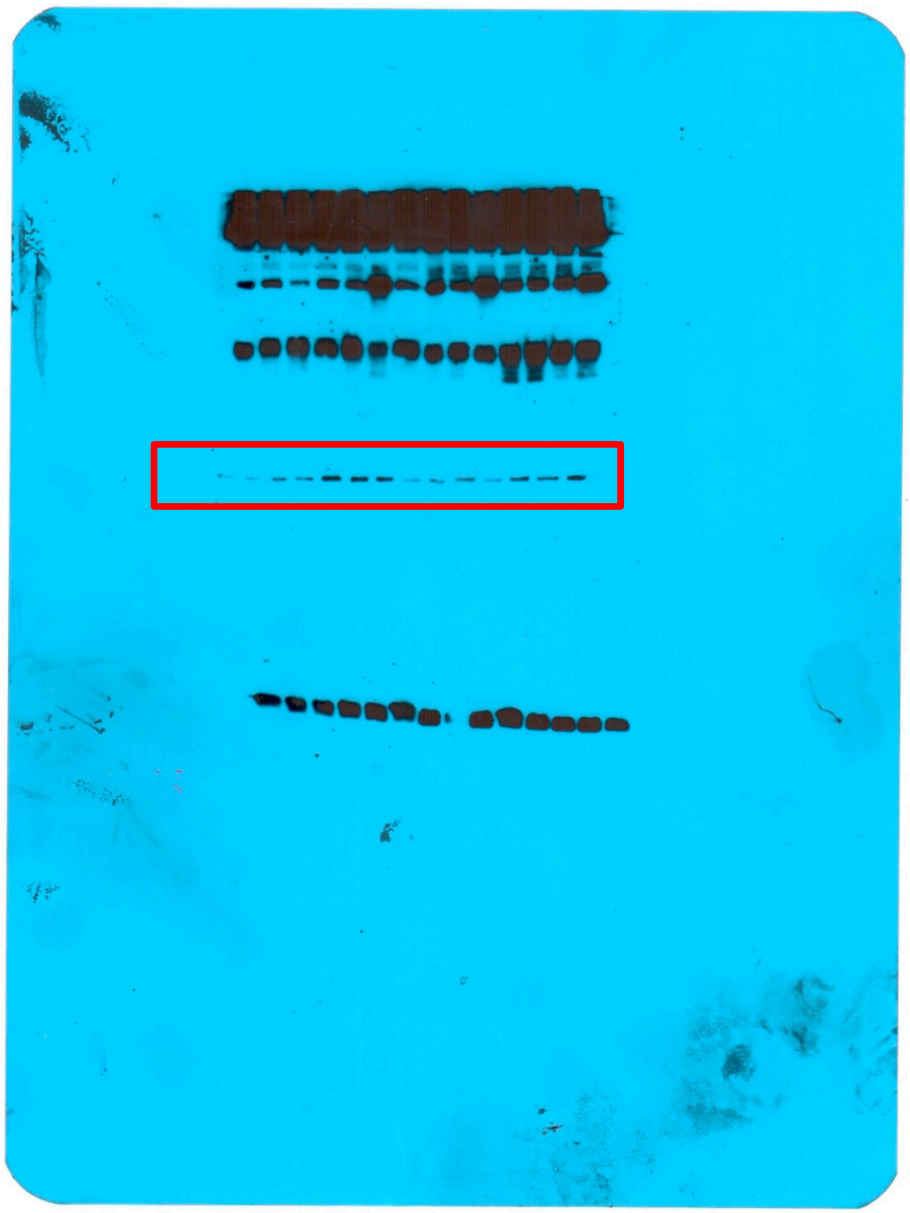

Supplement: Supplementary file 1 [file nutrients-15-04711-s001.zip › Supplemental data 2.pdf]
